# Suprachiasmatic nucleus-dependent and independent outputs driving rhythmic activity in hypothalamic and thalamic neurons

**DOI:** 10.1186/s12915-020-00871-8

**Published:** 2020-09-30

**Authors:** Court Harding, David A. Bechtold, Timothy M. Brown

**Affiliations:** Centre for Biological Timing, Faculty of Medicine, Biology and Health, University of Manchester, Manchester, UK

**Keywords:** Electrophysiology, Circadian, Paraventricular nucleus, Subparaventricular zone, Channelrhodopsin

## Abstract

**Background:**

Daily variations in mammalian physiology are under control of a central clock in the suprachiasmatic nucleus (SCN). SCN timing signals are essential for coordinating cellular clocks and associated circadian variations in cell and tissue function across the body; however, direct SCN projections primarily target a restricted set of hypothalamic and thalamic nuclei involved in physiological and behavioural control. The role of the SCN in driving rhythmic activity in these targets remains largely unclear. Here, we address this issue via multielectrode recording and manipulations of SCN output in adult mouse brain slices.

**Results:**

Electrical stimulation identifies cells across the midline hypothalamus and ventral thalamus that receive inhibitory input from the SCN and/or excitatory input from the retina. Optogenetic manipulations confirm that SCN outputs arise from both VIP and, more frequently, non-VIP expressing cells and that both SCN and retinal projections almost exclusively target GABAergic downstream neurons. The majority of midline hypothalamic and ventral thalamic neurons exhibit circadian variation in firing and those receiving inhibitory SCN projections consistently exhibit peak activity during epochs when SCN output is low. Physical removal of the SCN confirms that neuronal rhythms in ~ 20% of the recorded neurons rely on central clock input but also reveals many neurons that can express circadian variation in firing independent of any SCN input.

**Conclusions:**

We identify cell populations across the midline hypothalamus and ventral thalamus exhibiting SCN-dependent and independent rhythms in neural activity, providing new insight into the mechanisms by which the circadian system generates daily physiological rhythms.

## Background

Most aspects of mammalian physiology and behaviour exhibit pronounced daily variations under control of an internal circadian timing system [1, 2]. While there are local clocks across the brain and body that contribute to these processes, the maintenance of robust whole animal physiological timing and its coordination with environmental cycles relies on a master clock located in the hypothalamic suprachiasmatic nuclei (SCN) [3–5]. Hence, the molecular clock in SCN neurons drives circadian variation in membrane excitability and electrical output, and retinal inputs to the SCN align these neuronal oscillators to transmit high amplitude timing signals to their downstream targets [6, 7].

By contrast with our relatively advanced understanding of timekeeping within the SCN, the mechanism by which this central clock communicates downstream timing information is less well-understood. SCN neurons are primarily (if not exclusively) GABAergic and send direct neural outputs to a restricted set of midline nuclei including the subparaventricular zone (SPZ) and paraventricular nucleus of the hypothalamus (PVN) and paraventricular nucleus of the thalamus [8–11]. In vivo recordings of population activity from these SCN target regions in nocturnal rodents reveal circadian rhythms characterised by high firing during the animals’ active period [12–16]. Since neural activity within the SCN is high during the day (i.e. the rest period for nocturnal animals), a parsimonious model suggests inhibitory output from the SCN drives antiphase rhythms in downstream target structures. However, the extent to which the observed neuronal activity rhythms are secondary to (rather than causative of) daily variations in animal behaviour (e.g. locomotor activity, feeding/drinking) remains uncertain. Indeed, recordings from these target structures ex vivo [17–20] or in anaesthetised animals [21] suggest neural activity does not globally vary in antiphase to that of the SCN.

It is also now clear that SCN-dependent control of physiological timing is likely much more complex than originally envisioned. In mice, there are at least 5 different classes of SCN neuron based on the co-expression of various neuropeptides, with at least some of these exhibiting differentially phased patterns of activity [19, 22]. For example, we recently demonstrated that SCN cells expressing vasoactive intestinal polypeptide (VIP) exhibit peak firing during the mid to late day and drive inhibitory (GABAergic) responses in downstream target neurons to supresses their firing during that portion of the circadian cycle [23]. However, in the same experiments, we found that many neighbouring neurons also exhibited 24-h rhythms in neural activity, yet with peaks in activity spread across all possible phases of the circadian cycle. It is not yet clear whether such diversity in neuronal activity rhythms reflects input from distinct classes of SCN neurons providing inputs with different timing and/or neurochemical basis (i.e. excitatory rather than inhibitory [24–27]), or instead stems from local clock function (reviewed in [28]).

Here then, we set out to better understand how SCN-derived signals influence neural activity in target sites across the midline hypothalamus and ventral thalamus. To this end, we employ a combination of large scale multielectrode recording in acute ex vivo slice preparations, optogenetic and electrical stimulation of the SCN and pharmacological and physical (SCN removal) manipulation to define the nature of SCN influences on- and the phenotype of-downstream target neurons.

## Results

### Identification of SCN target neurons in the midline hypothalamus and ventral thalamus

**Fig. 1:**
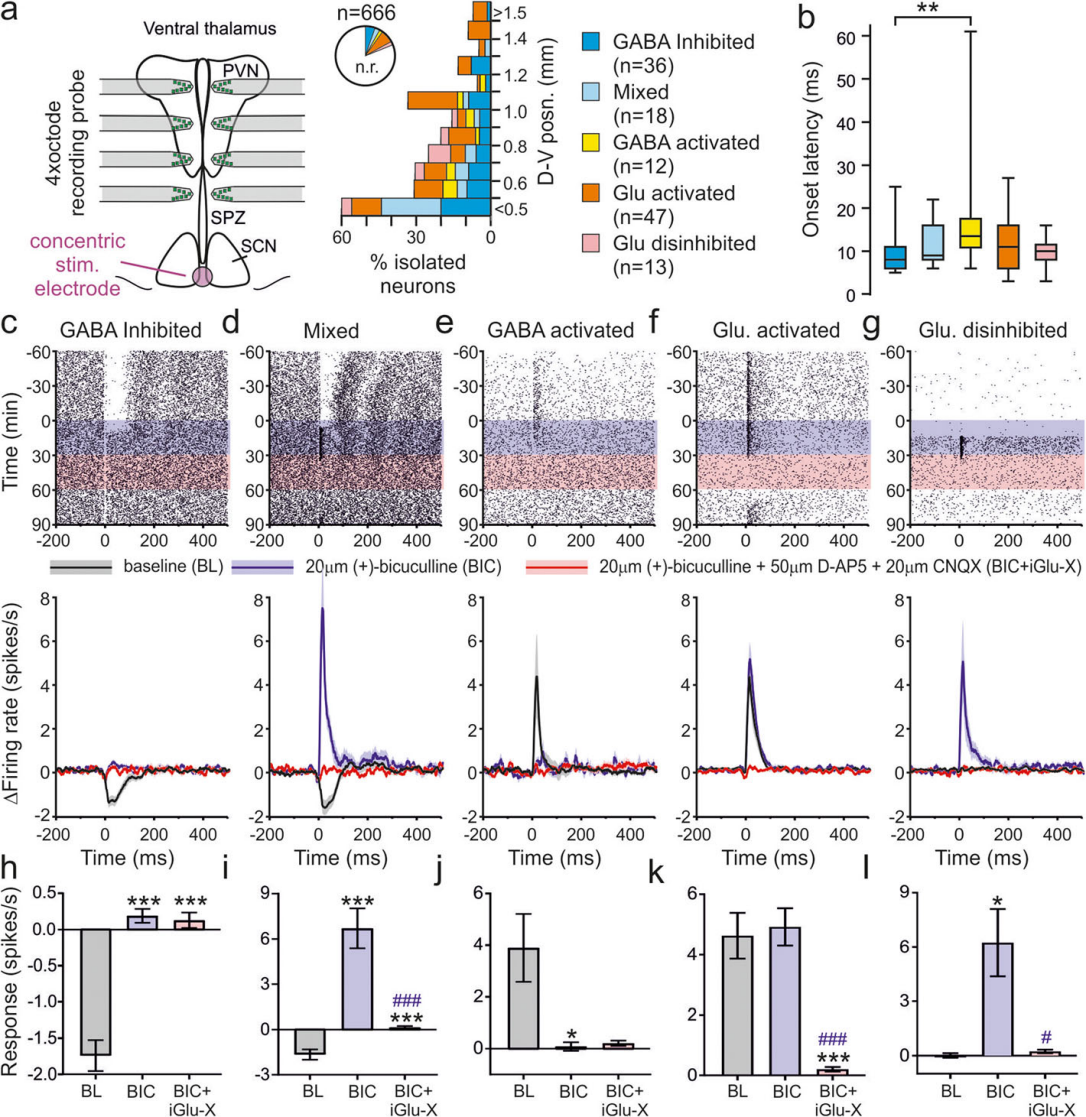
Stimulation of the SCN region drives a variety of GABA and glutamate driven responses in downstream target neurons. **a** Schematic of typical ex vivo recording and stimulation configuration (left) and proportions of isolated neurons exhibiting various classes of response to electrical stimulation as a function of dorsal-ventral location (right; n.r. indicates non-responsive cells). **b** Box and whisker plot showing distribution of response latencies (time to response onset) for various responsive cell classes. Data analysed by mixed-effects linear model (*F*_4,120_ = 3.3, *P* = 0.01) with Sidak’s post-tests. **c**–**g** Peri-stimulus raster plots from example cells (top) and mean ± SEM change in firing across the population of cells subdivided based on their responsive to electrical stimulation and its sensitivity to antagonists of fast ionotropic GABA and glutamate signalling. **h**, **i** Mean ± SEM response (peak change in firing occurring within 100 ms post stimulation) for the five identified classes of neurons (corresponding to those illustrated in **c**–**g**) under baseline and following antagonist treatment. Data analysed by mixed-effects linear model (**h** *F*_2,53.0_ = 37.3, *P* < 0.001; **i** *F*_2,18.3_ = 25.8, *P* < 0.001; **j** *F*_2,11.7_ = 4.2, *P* = 0.04; **k** *F*_2,46.7_ = 45.5, *P* < 0.001; **l** *F*_2,15.6_ = 6.3, *P* = 0.01) with Sidak’s post-tests. *, **, *** = *P* < 0.05, *P* < 0.01 and *P* < 0.001 vs. baseline. #, ### indicates *P* < 0.05 and *P* < 0.001 vs. BIC alone. Raw data values used for statistical analysis can be found in Additional file 8.

To obtain a relatively unbiased picture of how SCN electrophysiological output influences neuronal activity in downstream target nuclei, we first evaluated responses to electrical stimulation of the SCN region. Accordingly, we performed multielectrode (64 channel) recordings from the SPZ, PVN and ventral thalamus (and in a few cases also the SCN) while delivering intermittent current pulses via a concentric stimulating electrode centred between ventromedial portions of the paired SCN (Fig. 1a, Additional file 1: Fig. S1a). This position was chosen to allow us to stimulate the SCN in both hemispheres simultaneously, while minimising the risk of stimulating neurons located in adjacent parts of the hypothalamus. To facilitate subsequent optogenetic circuit mapping, experiments were performed in slices derived from mice where cre-dependent channelrhodopsin 2 was directed to VIP-expressing (VIP^+/cre^; Ai32^+/−^, *n* = 20) or all GABAergic cells (GAD2^+/cre^; Ai32^+/−^, *n* = 40), as well as cre-negative Ai32^+/−^ littermates (*n* = 13) [18, 19, 29–32]. Since there were no overt differences in the prevalence or nature of responses to electrical stimulation of the SCN identified across these genotypes, we combined the relevant data for initial analysis.

To confirm that our experimental approach effectively activated SCN neurons, we first analysed data from a subset cells recorded within the SCN itself (*n* = 23) or nearby regions of the SPZ (*n* = 59 cells within 600 μm of the stimulation site). Of note, a substantial proportion of SCN neurons (*n* = 17/23) exhibited robust increases in firing following local electrical stimulation that, in all but 1 cell, persisted following treatment with 20 μm (+)-bicuculline and 50 μm D-AP5/20 μm CNQX (BIC+iGluX) to block fast ionotropic GABA and glutamate receptors (Additional file 2: Fig. S2a-c). By contrast, no cells outside the SCN responded under these conditions. Thus, our electrical stimulation approach provided widespread and selective activation of SCN output, engaging ~ 70% cells within a radius of 325 μm from centre of the stimulation site (Additional file 2: Fig. S2d).

We next then examined in more detail the response of extra-SCN cells to electrical stimulation of the SCN region. From the experiments outlined above, we were able to isolate the activities of 666 individual neurons in total across the SPZ, PVN and ventral thalamus. The proportions of responding neurons varied strongly as a function of distance from the SCN, with relatively high proportions of responding neurons in SPZ and lower proportions at the more dorsal regions we recorded from (Fig. 1a). Consistent with previous investigations of SCN output pathways in the rat [25, 27], among those responding neurons, we found subsets of cells that exhibited reproducible decreases (*n* = 54) or increases (*n* = 59) in firing following electrical stimulation of the SCN. Although mouse SCN neurons are considered to be primarily, if not exclusively, GABAergic [8, 22], electrical stimulation of the SCN region has previously been reported to engage glutamatergic output [33]. Accordingly, we probed the neurochemical basis of the responses identified here by bath applying BIC followed by co-application with ionotropic glutamate receptor antagonists (iGlu-X), as above. On the basis of changes in response to SCN electrical stimulation and following antagonist treatments, this allowed us to identify five basic classes of target neurons (Fig. 1a–g).

Among those cells that reduced spike output following electrical stimulation under baseline conditions, in the majority of cases (*n* = 36/54), BIC treatment completely abolished responses, confirming a GABAergic origin (Fig. 1c, h; termed ‘GABA inhibited’). Interestingly, however, in the remaining subset (*n* = 18/54), inhibitory responses were replaced by robust increases in firing following BIC treatment which were subsequently abolished under BIC+iGlu-X (Fig. 1d, i). Hence, electrical stimulation drove both GABAergic and glutamatergic input to such cells with ‘mixed’ responses, albeit with inhibitory GABAergic responses dominating under baseline conditions. Both types of inhibitory cells exhibited similar response kinetics (Fig. 1b) and their prevalence decreased as a function of distance from the SCN (Fig. 1a; *χ*^2^ test, *P* < 0.001), in keeping with previously reported variations in the density of SCN output projections [8, 11].

By contrast to the above, few of the cells that exhibited excitatory responses to electrical stimulation under baseline conditions were sensitive to BIC (Fig. 1e, j; *n* = 12/59 cells). Interestingly, across the modest number of ‘GABA activated’ cells identified here, we found response latencies were significantly slower than for GABA inhibited cells (Fig. 1b, Sidak’s post-test, *P* = 0.006). This suggests such responses may arise via a multisynaptic mechanism (e.g. due to a decrease in inhibitory input from ‘GABA inhibited’ cells), consistent with our identification of cells with similar properties following optogenetic stimulation of SCN VIP cells [19]. However, in the majority of cases where we observed excitatory responses (*n* = 47/59), these persisted under BIC but were abolished under BIC+iGlu-X, indicating a glutamatergic origin (Fig. 1f, k; termed ‘Glu. activated’). Of note, BIC treatment also revealed an additional population of cells (*n* = 13) that exhibited glutamatergic excitatory responses but entirely lacked any overt response to electrical stimulation under baseline conditions (Fig. 1g, l; termed ‘Glu. Disinhibited’). Across these groups of cells with excitatory glutamatergic responses, kinetics were broadly similar to those with inhibitory responses (Fig. 1b), and these cells were more commonly found in the SPZ/PVN than ventral thalamic regions (*n* = 65/532 vs. 7/134 respectively, Fisher’s exact test, *P* = 0.02).

To account for the possibility that the prevalence or nature of target cell responses to SCN stimulation varied in a circadian manner; subsets of the experiments described above were performed in slices prepared either at the beginning (ZT1-3) or end (ZT10-12) of the animals light phase (*n* = 29 and *n* = 44 slices, respectively). While most of the response types described above were found in similar proportions between these two time-points ‘Glu. activated’ cells were more commonly identified during early day recordings (Additional file 3: Fig. S3a). We did not detect any significant variation in stimulus-evoked responses or baseline firing rates under the various treatment conditions for any cell type (Additional file 3: Fig. S3b-f). We did, however, note a trend towards reduced spontaneous firing rates and response amplitudes (prior to antagonist treatment) for GABA inhibited cells recorded during early day vs. early night (Additional file 3: Fig. S3b). This observation would be consistent with the notion that the daytime increase in spontaneous GABAergic output from the SCN drives circadian variation in the activity of these GABA inhibited cells (investigated in more detail below).

Our finding that electrical stimulation evokes GABAergic, primarily inhibitory, responses in a subset of neurons across the SPZ, PVN and ventral thalamus is in keeping with known SCN neurochemistry [8, 10, 22]; however, the presence of many cells exhibiting glutamatergic responses is surprising. Indeed, while there is certainly prior evidence that stimulation of the SCN region can evoke glutamatergic responses [25, 27, 33], clear anatomical evidence for a population of glutamatergic cells in the mouse SCN is lacking [10]. Alternatively then, glutamatergic responses could instead originate with stimulation of glutamatergic neurons found lateral to the SCN [10] or via retinal afferents that pass through the nucleus [34–36].

**Fig. 2:**
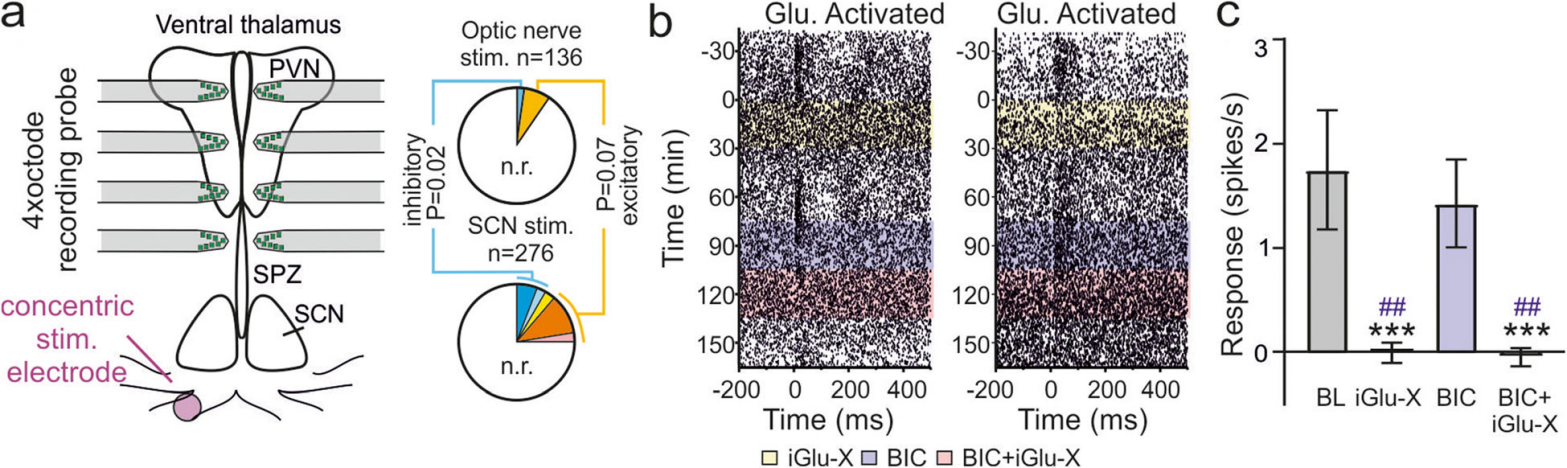
Optic nerve stimulation evokes primarily excitatory responses to SCN target regions. **a** Schematic of the recording configuration for experiments using optic nerve stimulation (left) and pie charts illustrating proportions of responding cells (n.r. indicates no response) following optic nerve stimulation and SCN stimulation performed at an equivalent time of day (slices prepared ZT1-3). Data analysed by *χ*^2^ test (*P* = 0.004) followed by Fisher’s exact tests for cells exhibiting inhibitory/GABAergic and excitatory/glutamatergic responses. **b** Peri-stimulus raster for two representative cells exhibiting excitatory responses to optic nerve stimulation before and after treatment with inotropic glutamate (iGlu-X; 50 μm D-AP5 and 20 μm CNQX) and/or GABA receptor antagonists (BIC; 20 μm (+)-bicuculline). **c** Mean ± SEM responses of neurons exhibiting excitatory responses to optic nerve stimulation (*n* = 10) in the presence and absence of inotropic glutamate and/or GABA receptor antagonists. Data analysed by mixed-effects linear model (*F*_3,33.3_ = 10.3; *P* < 0.001) with Sidak’s post-tests. *** = *P* < 0.001 vs. baseline (BL). ## = *P* < 0.01 vs. BIC. Raw data values used for statistical analysis can be found in Additional file 8.

To evaluate the latter possibility, we next performed a similar set of recordings to those described above but with the stimulating electrode placed on one of the optic nerves (Fig. 2a, Additional file 1: Fig. S1b). Across 9 slices (prepared during early day), we identified 10 cells that exhibited excitatory responses and 3 cells that displayed stimulus-driven decreases in firing in response to optic nerve stimulation. These responses were primarily observed at recording sites contralateral to the stimulated optic nerve (8/10 excitatory and 2/3 inhibitory), in line with prior anatomical observations of retinofugal projections [34–36]. Excitatory responses were reliably blocked by iGlu-X treatment (both alone and in combination with BIC) but were not blocked by BIC alone (Fig. 2b, c). Moreover, the proportion of cells displaying these glutamatergic activations was not statistically different to that observed following SCN stimulation (including both Glu. activated and disinhibited subtypes) in slices prepared at the same time of day (Fig. 2a; Fisher’s exact test, *P* = 0.07). By contrast, the proportion of cells exhibiting inhibitory responses following optic nerve stimulation was significantly less than we encountered following SCN stimulation (Fig. 2a, Fisher’s exact test, *P* = 0.02), nor did we observe any cells exhibiting GABAergic excitations. For one of the three neurons that was inhibited following optic nerve stimulation, the decrease in firing was blocked by iGlu-X treatment alone, suggesting that response was secondary to glutamatergic stimulation of the SCN (Additional file 4: Fig. S4a,c). The same was never observed for cells showing inhibitory responses following SCN stimulation (Additional file 4: Fig. S4b,d; *n* = 7 GABA inhibited and *n* = 4 mixed tested). It remains unclear whether responses of the remaining two neurons exhibiting GABA-mediated inhibitions following optic nerve stimulation reflected some minimal direct activation of the SCN or, perhaps, a role for the recently discovered subset of GABAergic intrinsically photosensitive retinal ganglion cells (ipRGCs) [37]. In either case, since optic nerve stimulation readily evokes excitatory glutamatergic responses in downstream neurons, we conclude that the occurrence of such responses following SCN stimulation at least primarily reflects stimulation of the retinal afferents that pass through the SCN on route to those regions [34–36].

**Fig. 3:**
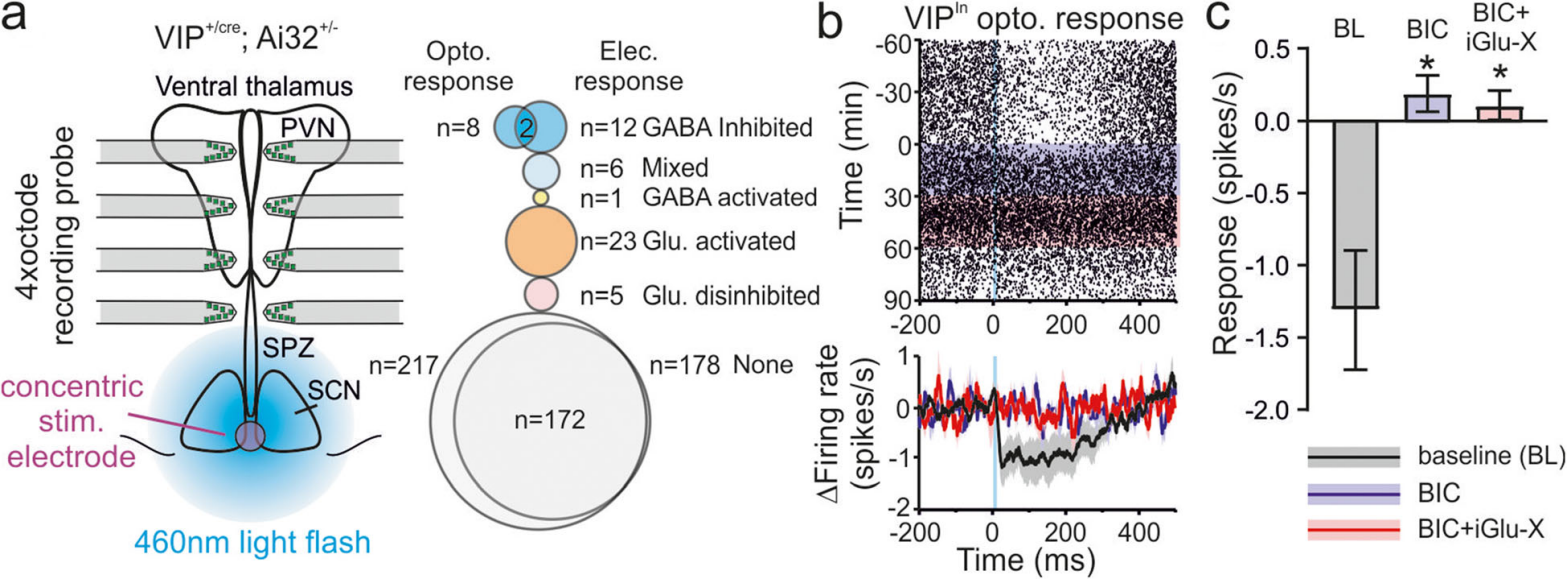
Inhibitory responses driven by SCN electrical stimulation arise primarily via non-VIP expressing neurons. **a** Left shows a schematic of the recording configuration for experiments using SCN electrical and optogenetic stimulation in VIP^+/cre^; Ai32^+/−^ slices, with 465 nm blue light flashes illuminating a region > 500 μm diameter centred on the SCN (see the “Methods” section). Right plot illustrates proportions of responding cells and their overlap following optical and electrical stimulation (bubble size proportional to population sizes). **b** Top panel shows peri-stimulus raster for a representative cell exhibiting inhibitory responses to optogenetic stimulation under baseline conditions and following GABA_A_ receptor blockade (BIC; 20 μm (+)-bicuculline) in the absence and presence of ionotropic glutamate receptor antagonists (iGlu-X; 50 μm D-AP5 and 20 μm CNQX). Lower panel shows mean ± SEM change in firing across the population of responding cells (*n* = 8) under the various conditions. **c** Mean ± SEM responses of neurons exhibiting inhibitory responses to optogenetic stimulation before and after ionotropic GABA/glutamate receptor blockade. Data analysed by mixed-effects linear model (*F*_2,9.9_ = 6.1; *P* = 0.02) with Sidak’s post-tests. * = *P* < 0.05. Raw data values used for statistical analysis can be found in Additional file 8.

We previously reported that optogenetic stimulation of SCN VIP cells drives inhibitory (GABA-mediated) responses in a subset of neurons across the SPZ, PVN and ventral thalamus [19]. To determine the extent to which the inhibitory responses observed here reflect activation of GABAergic output from VIP neurons, in a subset of experiments (*n* = 20 VIP^+/cre^;Ai32^+/−^ slices), we combined SCN electrical stimulation with optogenetic VIP-cell stimulation (Fig. 3a). As reported previously, wide-field blue light flashes applied over the SCN drove robust inhibitory responses in a subset of neurons in downstream target regions (Fig. 3a, *n* = 8) that were abolished under BIC treatment (Fig. 3b, c). This population of VIP-innervated (VIP^In^) cells only partially overlapped with those exhibiting inhibitory responses to electrical stimulation. Indeed, only 2 of 12 cells identified in these experiments with inhibitory responses and 0 of 6 cells with mixed responses to electrical stimulation also showed inhibitory responses to optogenetic stimulation (Fig. 3a). The optogenetic approach employed here robustly activates VIP cells throughout the SCN [19]; therefore, a substantial proportion of the SCN output revealed by electrical stimulation must come from non-VIP cells. Interestingly, however, we also found a number of VIP^In^ cells (*n* = 6/8) that did not display any overt responses to SCN electrical stimulation. Thus, the electrical stimulation approach employed here also, to some extent, underestimates the true proportion of downstream target neurons receiving SCN input.

To probe this possibility further, we next performed similar experiments in slices from animals where ChR2 expression is directed to GAD2-expressing cells, thereby targeting all GABAergic neurons throughout the SCN (GAD2^+/cre^; Ai32^+/−^). In pilot experiments, we found that wide-field optogenetic stimulation (delivered as above for VIP cells) resulted in direct activation of many neurons outside the SCN. Accordingly, to selectively target just SCN GABAergic neurons, we delivered local illumination within one SCN hemisphere via a finer (105 μm core) fibre attached to a penetrating recording electrode while recording from target neurons of the same hemisphere via a second electrode (Additional file 5: Fig. S5a; *n* = 16 slices). As expected, the majority of SCN neurons detected in these experiments (*n* = 18/21) exhibited robust excitatory responses to optogenetic stimulation that were unaffected by treatment with ionotropic glutamate and GABA receptor blockers (Fig. S5b,c). We also found a small subset of cells in downstream target regions (*n* = 5/115) that exhibited inhibitory, GABA-mediated responses, to optogenetic stimulation (Additional file 5: Fig. S5b,c). By comparison with selective stimulation of SCN VIP cells, a nominally greater proportion of these ‘GABA^In^’ cells also exhibited inhibitory responses to electrical stimulation of the SCN (*n* = 3/5). However, this approach also failed to evoke detectable responses in a subset of neurons that exhibited GABA-mediated responses to more conventional stimulation (*n* = 5/8 cells), in keeping with the more local nature of the optogenetic stimulus.

**Fig. 4:**
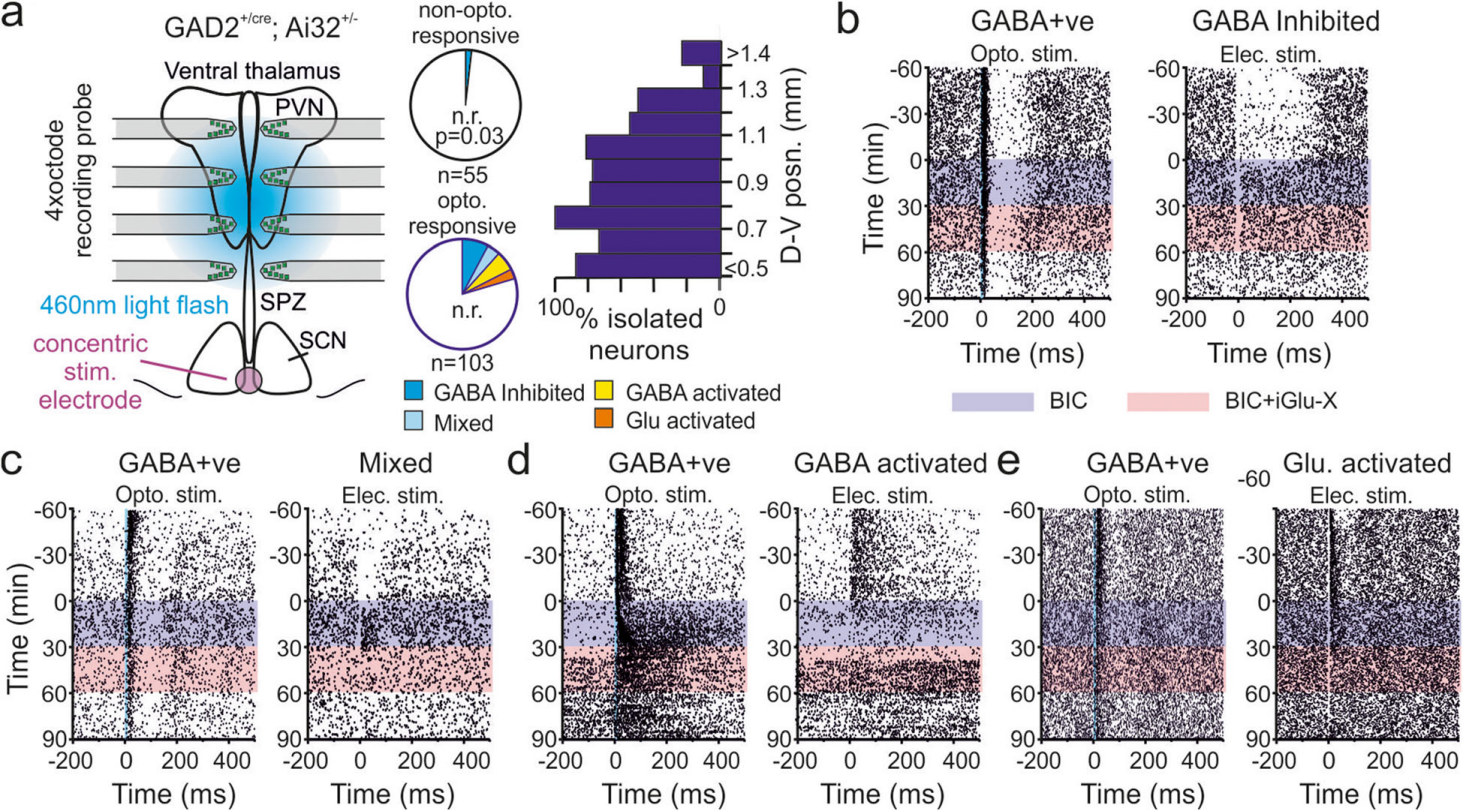
Neurons responding to electrical stimulation of the SCN region are primarily GABAergic in nature. **a** Left shows a schematic of the recording configuration for experiments using SCN electrical and optogenetic stimulation of target cells in GAD2^+/cre^; Ai32^+/−^ slices. Pie charts show proportions unresponsive (upper) and optogenetically activated (lower) target cells showing varying classes of response to electrical stimulation (n.r. indicates no response to electrical stimulation; difference in proportions analysed by *χ*^2^ test, *P* = 0.03). Right plot shows proportions of neurons showing optogenetic activation as a function of position on the dorsal-ventral axis. **b**–**e** Peri-stimulus rasters for representative cells showing response to optogenetic stimulation (left) and SCN electrical stimulation (right), under baseline conditions and following GABA_A_ receptor blockade (BIC; 20 μm (+)-bicuculline) in the absence and presence of ionotropic glutamate receptor antagonists (iGlu-X; 50 μm D-AP5 and 20 μm CNQX). Panels respectively show examples of neurons classed as GABA inhibited (**b**), Mixed (**c**), GABA activated (**d**) and Glu. activated (**e**) based on response to SCN electrical stimulation and effects of antagonist treatments. Raw data values used for statistical analysis can be found in Additional file 8.

Since electrical stimulation drove more widespread SCN output than targeted optogenetic stimulation, in subsequent experiments, we instead employed optogenetic approaches in GAD2^+/cre^; Ai32^+/−^ slices for identification of the neurochemical phenotype of SCN-target cells rather than driving downstream responses. Accordingly, in a final subset of recordings, we used wide field optogenetic stimulation of the SPZ, PVN and ventral thalamus alongside electrical stimulation of the SCN region to define which of those responding neurons were themselves GABAergic (Fig. 4a; *n* = 24 GAD2^+/cre^; Ai32^+/−^ slices). Consistent with the known neuroanatomy of the target regions [10], the majority of cells recorded from the SPZ and ventral portions of the PVN (~ 80%; Fig. 4a) exhibited robust activations following optogenetic stimulation that persisted in the presence of BIC+iGlu-X, indicating they were GABAergic (GAD2-expresing). By contrast, cells that lacked excitatory responses to optogenetic stimulation were less commonly encountered in recordings from more dorsal sites (Fig. 4a; PVN and, especially, the ventral thalamus) consistent with the greater proportion of glutamatergic neurons found in those regions [10]. Most notably, however, among cells that responded to electrical stimulation of the SCN region, all but one, also exhibited robust direct optogenetic activation (*n* = 8/9 GABA inhibited, Fig. 4b; *n* = 4/4 Mixed, Fig. 4c; *n* = 6/6 GABA activated, Fig. 4d; *n* = 3/3 Glu. Activated, Fig. 4e). Indeed, the proportions of neurons exhibiting responses to SCN electrical stimulation were significantly greater among optogenetically responsive vs. non-responsive cells (Fig. 4a; *χ*^2^ test *P* = 0.03). Thus, SCN (and retinal) output to the recorded regions almost exclusively targets GABAergic neurons.

### Circadian rhythmicity in hypothalamic and ventral thalamic targets of SCN and retinal input

**Fig. 5:**
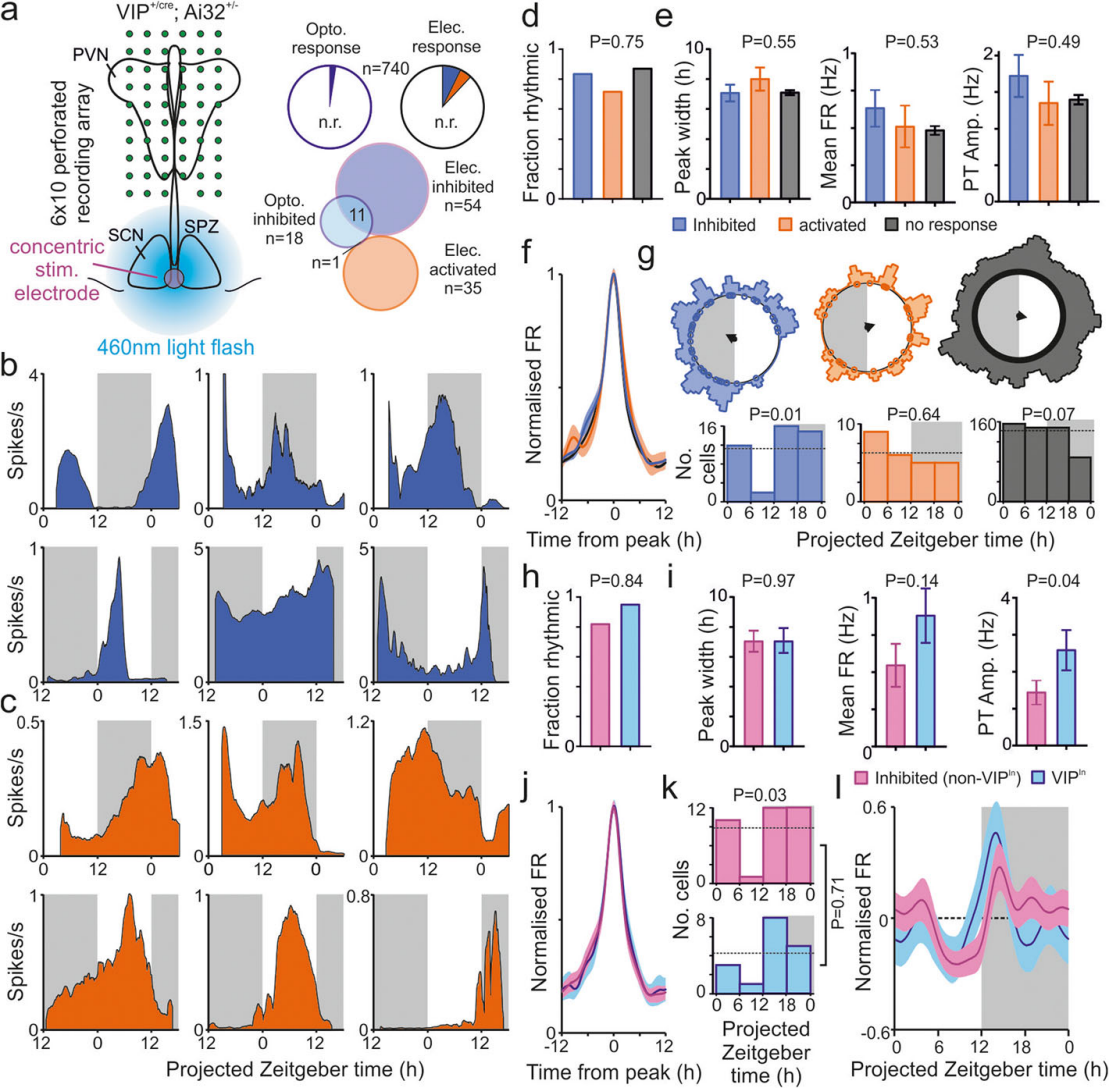
Inhibitory SCN input is associated with circadian firing patterns with peak activity during the night or early day. **a** Schematic of the recording/stimulation configuration for long-term monitoring of neural activity in SCN target regions of VIP^+/cre^; Ai32^+/−^ slices (left) and proportions of cells responding to optogenetic/electrical stimulation (n.r. indicates no response). **b**, **c** Spontaneous firing rates of representative neurons that were inhibited (**b**) or activated (**c**) by electrical stimulation of the SCN region, from slices prepared during early (upper panels) or late (lower panels) projected day. Rightmost cells from **b** were also inhibited by SCN optogenetic stimulation (VIP^In^). **d** Proportions of neurons with inhibition, activation or no response to SCN electrical stimulation (*n* = 54, 35 and 651 respectively) that displayed evidence of circadian variation. Data analysed by *χ*^2^ test. **e** Mean ± SEM duration of firing rate peak (left), 24 h average (mid) and peak-trough amplitude for rhythmic neurons showing inhibition, activation or no response to SCN electrical stimulation (*n* = 45, 25 and 566 respectively). Data analysed by mixed-effects linear model (left: *F*_2,628_ = 0.61, *P* = 0.55; mid: *F*_2,625_ = 0.63, *P* = 0.53; right: *F*_2,624_ = 0.72, *P* = 0.49). **f** Mean ± SEM normalised, peak-aligned, 24 h firing profiles for populations in **e**. **g** Rayleigh vector plots showing distributions of peak times for rhythmic neurons (outer histogram smoothed with Gaussian, SD = 15 min; individual neurons represented by inner dots). Lower panels show cells counts in 6 h bins, compared against a uniform distribution by *χ*^2^ test. **h**–**k** As in **d**–**g** but for VIP^In^ cells (*n* = 17/18 classed as rhythmic) vs. those showing inhibitory responses to electrical stimulation only (non-VIP^In^; *n* = 35/43 rhythmic), data in **i** analysed by mixed-effects linear model (left: *F*_1,43.6_ = 0.002, *P* = 0.97; mid: *F*_1,47.1_ = 2.22, *P* = 0.14; right: *F*_1,44.5_ = 4.65, *P* = 0.04). **l** Mean ± SEM normalised firing rate of the populations in **i**–**k** as a function of projected Zeitgeber time. Raw data values used for statistical analysis can be found in Additional file 8.

Having identified neurons across the SPZ, PVN and ventral thalamus receiving input from the SCN and/or retina, we next investigated in more detail whether such influences were associated with specific circadian patterns in spontaneous neural activity in the recipient neurons. To obtain stable long term (> 26 h) recordings from individual target neurons, we performed perforated multi-electrode array (pMEA) recording in VIP^+/cre^; Ai32^+/−^ slices (*n* = 15 prepared during early day and *n* = 18 during late day). We then combined both electrical and optogenetic stimulation of the SCN region to distinguish VIP^In^ cells from those cells that received inhibitory input from other classes of SCN neurons (Fig. 5a).

Consistent with our experiments using penetrating electrodes, electrical stimulation of the SCN region reliably identified subsets of target neurons displaying inhibitory (*n* = 54/740) and excitatory responses (*n* = 34/740). So as not to disrupt ongoing rhythmic activity during these long-term recordings, we did not apply antagonist treatments to determine the neurochemical basis of such responses here. Nonetheless, based on the data presented above, the former group (presumably including both GABA inhibited and Mixed subtypes) reflects those cells receiving strong GABAergic input from the SCN, while the latter group will primarily comprise those receiving excitatory glutamatergic input from the retina. We suspect this includes ‘Glu. Disinhibited’ cells since these longitudinal recordings revealed that synaptically driven responses were not always evident for the full duration of our recordings, despite continued spontaneous firing (Additional file 6: Fig. S6a). Across the populations of cells that were activated or inhibited by SCN electrical stimulation, response amplitudes did not vary in a consistent manner as a function of time of day or time since start of recording, however (Additional file 6: Fig. S6b).

We next examined the spontaneous firing profiles of neurons responsive to electrical stimulation of the SCN region (Fig. 5b,c). The majority of both inhibited (*n* = 45/54) and activated neurons (*n* = 25/35) displayed evidence of circadian variation in firing (see methods), as did cells that lacked responses to electrical stimulation (*n* = 566/651; Fig. 5d, *χ*^2^ test, *P* = 0.75). Further, among rhythmic cells in each class, the duration of the high firing epoch, 24 h mean and peak-trough amplitude of the firing rate rhythm were all statistically equivalent (Fig. 5e). Importantly, however, while the basic circadian waveforms of neurons in each class were virtually identical (Fig. 5f), the timing of peak firing showed substantial variation across the groups (Fig. 5g).

Among cells exhibiting inhibitory responses to electrical stimulation there was a striking absence of peak firing across the mid-late projected day (Fig. 5g; *χ*^2^ test, *P* = 0.01). The timing of peak firing was not overtly associated with anatomical location (Additional file 6: Fig. S6c) nor with time since start of recording (Additional file 6: Fig. S6e-f; *χ*^2^ test, *P* = 0.65). Thus, cells that receive inhibitory input from the SCN exhibit a broad but non-random distribution of phases, with peak firing during the night or early projected day. Analysis of the phase distributions of cells across slice preparations produced equivalent results, highlighting a significant variation when the phase of peak firing was expressed relative to prior LD cycle but not time since start of recording (Additional file 4: Fig. S6d,g).

By contrast, neither cells that were activated by electrical stimulation of the SCN region nor those that were unresponsive exhibited this property. Instead, the phases of peak firing for the latter two groups were almost evenly distributed across the day and night (Fig. 5f; Additional file 6: Fig. S6d). Further analysis indicated that for unresponsive (but not activated cells), the phasing of cellular rhythms exhibited a weak but significant clustering relative to time since start of recording (Additional file 6: Fig. S6e-g). Thus, it appears that that the acute slice recording procedure either induces or re-sets neuronal rhythms in a subset of neurons across the PVN, SPZ and ventral thalamus that lack input from the SCN or retina.

We next employed optogenetic stimulation to discriminate which of the recorded neurons received input from SCN VIP cells. As expected, a subset of neurons exhibited inhibitory responses to optogenetic stimulation (*n* = 18 VIP^In^ cells) that partially overlapped with those inhibited following electrical stimulation (Fig. 5a, Fig. S6a). However, the majority of cells that were inhibited by SCN electrical stimulation were non-VIP^In^ (*n* = 43/54). Nevertheless, in line with our characterisation of inhibited (primarily non-VIP^In^) cell populations above and previous analyses of VIP^In^ cells [19], VIP^In^ and non-VIP^In^ electrically inhibited cells were very similar (Fig. 5h–l). Hence, both groups exhibited similar basic circadian properties (Fig. 5h–j) and exhibited a broad but non-random distribution of phases (Fig. 5k, Additional file 6: Fig. S6d-g). We did, however, find that peak-trough amplitudes were significantly higher among VIP^In^ cells (Fig. 5i), consistent with our previous data indicating that SCN VIP cells exhibit particularly robust circadian firing rate rhythms [19].

### Reliance of hypothalamic and thalamic neuronal rhythms on SCN output

**Fig. 6:**
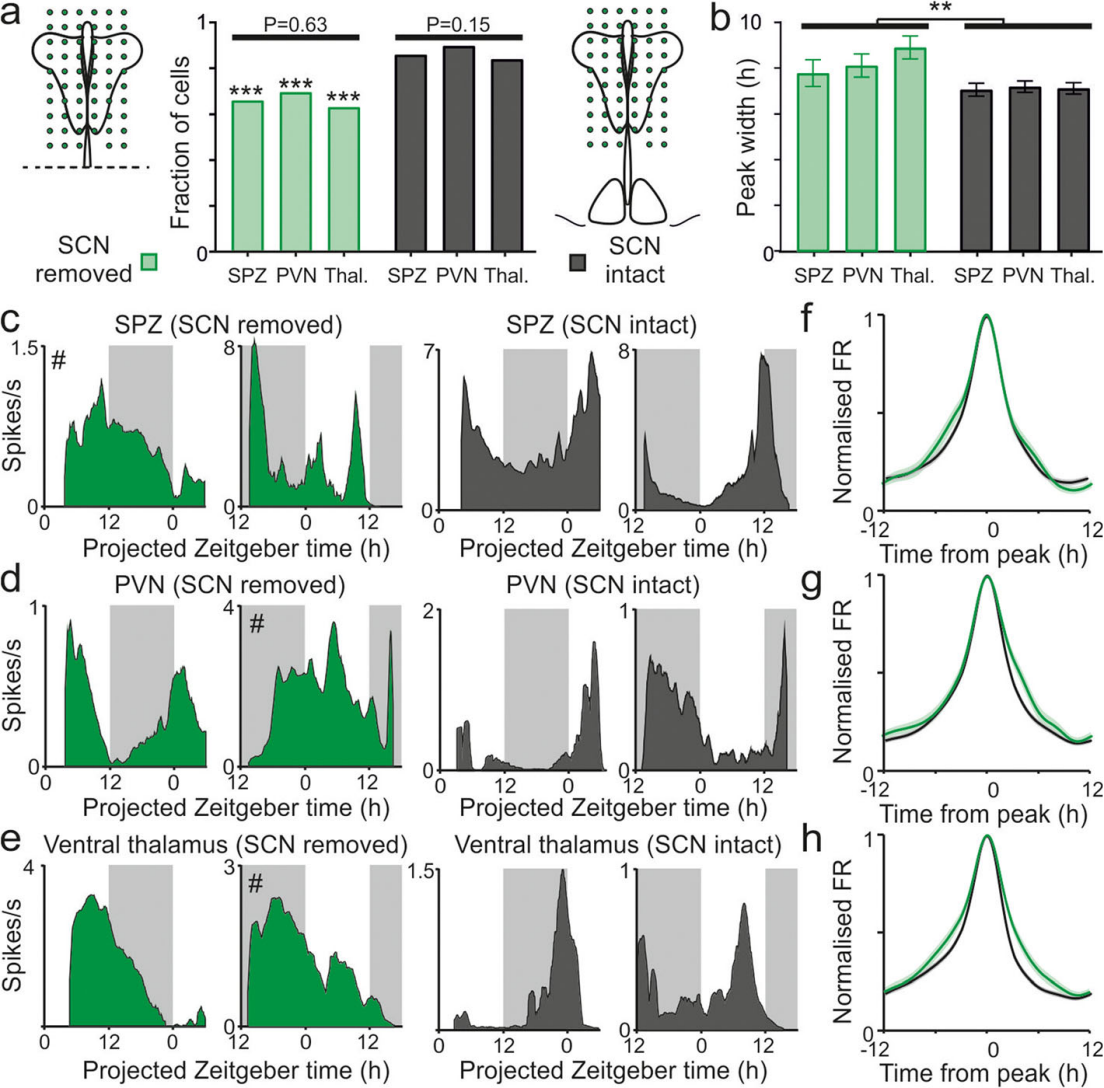
SCN-independent neuronal rhythms in subsets of hypothalamic and ventral thalamic neurons. **a** Proportions of rhythmic neurons detected in ex vivo recordings for the SPZ, PVN and ventral thalamus in slices lacking (*n* = 51/78, 58/84 and 75/120 respectively) and containing the SCN (*n* = 169/198, 231/259 and 236/283 respectively). **b** Mean ± SEM width of firing rate peak for neurons isolated from SCN target regions in slices lacking or containing the SCN. Data analysed by mixed-effects linear model (SCN: *F*_1,46_ = 8.63, *P* = 0.005; region: *F*_2,631_ = 0.65, *P* = 0.52; interaction: *F*_2,631_ = 0.56, *P* = 0.57). **c**–**e** Representative spontaneous firing profiles recorded from SPZ (**c**), PVN (**d**) and ventral thalamus (**e**) from slices prepared during early or late day and containing or lacking the SCN. Neurons that did not meet criteria for rhythmicity (see the “Methods” section) indicated by #. **f**–**h** Mean ± SEM normalised and peak aligned firing rate profiles for rhythmic neurons from SPZ (**f**), PVN (**g**) and ventral thalamus (**h**) in slices containing or lacking and SCN. Raw data values used for statistical analysis can be found in Additional file 8.

Our data indicate that inhibitory SCN output influences neuronal activity rhythms in a subset of neurons across the SPZ, PVN and ventral thalamus. However, we also identify many other neurons in those target regions that exhibit circadian modulations in firing, but lack observable responses to electrical/optogenetic stimulation of the SCN. To better understand the potential reliance of these oscillations on rhythmic output from the SCN, we next prepared slices equivalent to those used above but with the SCN region removed by scalpel cut prior to pMEA recording (Fig. 6a). We then compared the resulting data to that from equivalent recordings in slices containing the SCN (i.e. cells contributing to Fig. 5 regardless of response to stimulation).

We were still able to identify many neurons across the SPZ, PVN and ventral thalamus (*n* = 51/78, 58/84 and 75/120 respectively) that displayed evidence of circadian rhythmicity in the complete absence of the SCN (Fig. 6c–e). However, the proportion of neurons that passed our criteria for rhythmicity was significantly reduced in each region, relative to slice recordings with the SCN intact (Fig. 6a; *χ*^2^ tests, all *P* < 0.001). As expected from our analysis of cells that lack detectable SCN input (Fig. 5g and Additional file 6: S6d-g), across populations of cells from each anatomical region, the phase of peak firing was similar in SCN-removed and intact slices, being more closely related to time since the start of recording rather than projected time of day, particularly for SPZ cells (Additional file 7: Fig. S7a-c). We did, however, observe subtle changes in the nature of the rhythmic activity in the absence of the SCN. Specifically, while overall mean firing rates and rhythm amplitude were similar to those observed in SCN containing slices (Additional file 7: Fig. S7d-e), the circadian waveforms of neurons were broader in slices that lacked the SCN (Fig. 6b, f–h). This implies a further role for SCN-derived signals in shaping the circadian waveforms of at least some of those neurons that do not absolutely rely on the central clock to rhythms in spontaneous firing.

Collectively then, these data identify distinct classes of oscillatory neurons across the major SCN target regions in the SPZ, PVN and ventral thalamus. A subset of neurons rely on intact SCN projections to sustain circadian variation in firing ex vivo. By contrast, a more numerous group of cells express at least transient neuronal rhythmicity which does not require input from the SCN, and whose phase is seemingly unrelated to prior light history, suggesting rhythms in of at least some of this this latter group are induced or reset ex vivo.

## Discussion

Midline hypothalamic and thalamic nuclei form primary targets of SCN output and are well known to exhibit rhythms in gene expression and neuronal activity in vivo [12–16, 28]; however, the precise role of clock and light-dependent signals in shaping these rhythms has long remained unclear. Here, we identify populations of GABAergic neurons across known SCN target regions that receive GABAergic input from the SCN and/or retinal glutamatergic input. We show that cells receiving inhibitory input exhibit circadian variation in firing ex vivo, whose phasing reflects the animals’ prior light photoperiod, with peak firing occurring during the night or early projected day. This arrangement recapitulates that previously described for those neurons receiving input from SCN VIP cells [19], and we extend this finding here to show it is true also of those that receive input from non-VIP expressing SCN cells. Further, we show that removal of the SCN abolishes rhythms in a subset of SPZ, PVN and ventral thalamic neurons ex vivo, indicating that SCN projections directly drive rhythms in neural activity in ~ 20% of neurons across those regions. By contrast, we also show that many other neurons in midline hypothalamic and thalamic target regions exhibit circadian variations in neural activity that are largely independent of any SCN derived signal and whose phasing does not reliably reflect the animals’ prior photoperiod. Hence, rhythmic activity in this latter group appears to be driven by more labile local rhythmic processes that are reset ex vivo, consistent with previous ex vivo imaging and electrophysiological recordings of SCN-independent rhythmic activity in other hypothalamic regions [38].

We interpret inhibitory GABAergic responses following SCN stimulation as those that receive direct input from SCN cells, while the less frequently observed (and more sluggish) excitatory GABAergic responses as originating via a polysynaptic pathway (as suggested previously based on optogenetic stimulation [19]). In line with this view, SCN electrical stimulation did not noticeably activate cells outside the SCN and the resulting downstream inhibitory responses were rapid and insensitive to ionotropic glutamate receptor block alone (hence could not involve any intervening glutamatergic neuron). Moreover, it is noteworthy that equivalent inhibitory responses were only very rarely observed following optic nerve stimulation. Those later observations could, in principle, arise due to some minor degree of direct SCN stimulation occurring in the later experiments or perhaps reflect the action of the recently discovered subpopulation of GABAergic ipRGCs [37]. Nonetheless, given that GABAergic inhibitions were more than 5 times more commonly observed following SCN rather than optic nerve stimulation, we are confident that this population primarily reflects those receiving SCN input. Also, in line with this interpretation, spontaneous firing across this population exhibited a pronounced dip across the mid-late day (when SCN firing activity is high), consistent with that previously observed following optogenetic stimulation of SCN VIP cells [19].

Interestingly, among those cells exhibiting inhibitory responses to SCN stimulation, neuronal rhythms are characterised by comparatively brief epochs of high firing activity (on average ~ 7 h when firing was > 50% of maximum). Since SCN neurons themselves display a similar property [19, 39–41], it seems unlikely that circadian rhythmicity in the target cells are directly imposed by inhibitory input from a single SCN neuron. Convergent input from several SCN neurons with divergent phasing [19, 39–41] would more conceivably explain the fairly long epochs of low firing and shorter epochs of elevated firing in the identified target neurons. Such an explanation might also explain the rather considerable variation in phasing between identified SCN-target cells revealed here and previously [19]. Since many of those cells we identify based on responses to electrical stimulation of the SCN lack detectable input from VIP cells, any such convergent input cannot uniformly pool inputs across classes of SCN output cells. Naturally, it is also possible that cell-intrinsic and/or rhythmic activity in other local neurons influence the shape and/or phasing of neuronal rhythms among cells that receive input from the SCN. Indeed, there is ample evidence for local clock function in the regions examined [28, 42–44], and many cells that we identified retain rhythmic activity in the absence of the SCN.

Our estimates of the proportion of cells that rely on SCN input for rhythmicity (> 20%), based on comparison of SCN intact and ablated recordings, is somewhat higher than the proportions of SCN-target cells identified by direct electrical stimulation of the SCN (< 10% of neurons). This most likely reflects the fact that the electrical stimulation approach (where we used modest current pulses to avoid activating extra-SCN regions) does not robustly activate all SCN output neurons. Indeed, our direct data from local SCN recording and stimulation suggests our approach activates ~ 70% of SCN neurons. Broadly in line with this estimate, we found that ~ 50% of downstream neurons responding to optogenetic activation of VIP cells (across acute or long-term recordings) also displayed inhibitory responses to electrical stimulation of the SCN. We also note, however, that neither the electrical or optogenetic approaches employed here evoked responses that could be ascribed to neuropeptide signalling (i.e. all responses were blocked by a combination of GABA and glutamate receptor antagonists). There is certainly evidence that both VIP [45–47] and AVP [24, 48] act as SCN output signals. A lack of VIP-mediated responses is unsurprising given that, in mice, cognate receptors are almost entirely absent from the SPZ, PVN and ventral thalamic regions examined here [10, 49], and exogenous VIP application is without effect [19]. It is certainly possible that signalling via AVP and/or other SCN neuropeptides (e.g. prokineticin2 [50]) could contribute to driving rhythms our target cells but that this form of signalling is not strongly activated by the electrical stimulation approach used here.

One of the somewhat surprising features of the present work was our identification of a significant population of neurons across the SPZ, PVN and ventral thalamus that exhibited glutamatergic excitatory responses. Glutamatergic responses to SCN stimulation have been reported previously in rat PVN and thalamus [25, 27] and have been suggested as a mechanism for commissural communication in the mouse SCN [33]. Nonetheless, in keeping with the lack of anatomical evidence for a major population of glutamatergic neurons in the mouse SCN [10], our present data suggest that the glutamatergic responses originate from retinal afferents. Hence, we do not find evidence of any significant direct activation of cells outside the SCN following local electrical stimulation and glutamatergic (but not GABAergic responses) are strongly retained when we stimulate the optic nerve. In line with a retinal origin for such responses, tracing studies also reveal retinal projections to the target regions we examined, suggesting glutamatergic excitatory responses primarily derive from melanopsin-expressing ipRGCs [34–36]. Accordingly, electrophysiological recordings have previously revealed cells across these regions that display melanopsin-dependent changes in firing [21].

Interestingly, our analysis of circadian firing profiles across cells that display excitatory responses to electrical stimulation of the SCN indicates that, while such cells can sustain rhythms in neural activity ex vivo, these do not adopt a predictable phase with respect to prior light history. This group potentially includes the small number of cells exhibiting GABA-mediated increases in firing (believed to be polysynaptic in origin [19]); however, the great majority of this group are expected to receive retinal input. The lack of phase clustering among these cells further suggests that their rhythmic activity ex vivo is largely independent of an SCN-output signals. Thus, It seems likely that such ex vivo rhythms instead originate with cell-intrinsic or, at least local, oscillatory process. The rhythmic output from such cells would presumably be modulated by diurnal variations in light exposure in the intact animal [21], but it seems any influence on underlying circadian process, if present, is lost ex vivo.

In line with the above, it is possible that light exposure could acutely regulate some of those cells which we identify as receiving inhibitory input. As noted above, it is formally possible that a small subset of these receive input from GABAergic ipRGCs [37]. More significantly, however, many SCN neurons display acute changes in firing in response to light exposure [51–55] and in vivo recordings from the midline hypothalamus and ventral thalamus reveal a population of cells exhibiting acute light-driven decreases in firing, believed to arise via input from the SCN [21]. As a population, that latter group of cells display evidence of diurnal variation in firing rate with low firing during the mid-late day and high firing at night, which is consistent with the properties of cells identified here as receiving SCN input. We do, however, also find some evidence for cells that combine inhibitory input from the SCN and excitatory input from the retina (i.e. those with ‘mixed’ responses). Under our ex vivo recording conditions, the latter influence is only apparent following pharmacological blockade of GABA signalling but it is harder to predict how this arrangement would impact neuronal activity in the intact animal. For example, inhibitory input from the SCN could potentially serve to provide circadian modulation to the responses of such cells to environmental light and/or a combination of clock and light-dependent input might dictate the phase of cell-intrinsic clocks regulating the activity of such neurons.

Finally, it is worth considering in more detail the fairly large proportion of neurons that we identify which appear capable of sustained rhythms in neural activity in the absence of the SCN. Clearly, the presence of cells expressing components of the molecular clock across the midline hypothalamus and ventral thalamus [28, 42–44] provides a substrate to sustain such activity. By the same token, our data indicate that any such local clock activity does not retain its phase on removal from the animal, with at least a sizeable subset instead showing oscillations whose phase is related to time ex vivo. In the behaving animal, a wide range of self-generated and/or environmental signals could indirectly drive and/or synchronise rhythmic activity in such cells to produce the population-level antiphase rhythms exhibited by these SCN output regions in vivo [12–16]. Future studies employing targeted knockout of the molecular clock from neurons local to these regions will be informative as to whether the intrinsic capacity for rhythmicity in such cells plays important roles in controlling whole animal physiological rhythms.

## Conclusions

Collectively, our data provide new insight into the mechanisms by which the central clock communicates timing information to downstream neurons in brain sites responsible for physiological and behavioural control. Strikingly, only a subset of cells across the SPZ, PVN and ventral thalamus directly rely on SCN input to express rhythms in neuronal activity. By contrast, many other cells sustain rhythms in spontaneous firing, apparently independently of any SCN-derived signals. Since this more numerous group of cells do not retain information about time of day ex vivo, the coordinated nocturnal elevation in activity previously observed in in vivo recordings from SCN target regions [12–16] likely reflects a much less direct form of SCN-dependent control, secondary to clock-driven physiological and behavioural rhythms.

## Methods

### Animals

All animals were used in accordance with the Animals, Scientific Procedures, Act of 1986 (UK) and received both institutional ethics committee and UK Home Office approval. Experimental animals were generated by crossing founders from the Jackson Laboratory (ME, USA): VIP-IRES-Cre (JAX #010908) or GAD2-IRES-Cre (JAX #010802) mice [30] and mice bearing Cre-dependent ChR2-EYFP construct (Ai32; JAX #0102569) [29]. Experiments therefore employed animals (45–155 days old) where ChR2 was directed to VIP expressing (VIP^+/cre^; Ai32^+/−^) [19, 31, 32] or GABAergic neurons (GAD2^+/cre^; Ai32^+/−^, *n* = 40) [18] or cre-negative Ai32^+/−^ littermates as appropriate. Mice were housed in 12:12-h light/dark cycles in a temperature controlled environment (22 °C). Zeitgeber time (ZT) 0 was designated as time of lights-on and ZT12 as lights-off. Food and water were provided ad libitum.

### Electrophysiological recordings

#### Slice preparation

Mice were removed from the home cage at ZT0-1 or ZT11-12 and culled via cervical dislocation, followed by rapid extraction of the brain. Coronal slices (350 μm thickness) containing the mid-rostrocaudal extent of the SCN and the PVN were prepared using a 7000 smz-2 vibrating microtome (Campden Instrument, UK). For a subset of experiments (*n* = 9), brain slices were prepared such that a portion (~ 500 μm) of optic nerve was also retained. Slicing was performed in an ice cold cutting solution (4 °C, bubbled with 95% O_2_/5% CO_2_) of composition: 189 mM sucrose, 10 mM D-glucose, 26 mM NaHCO3, 3 mM KCl, 5 mM MgSO4, 0.1 mM CaCl2, 1.25 mM NaH2PO4. Slices were subsequently transferred to aCSF (oxygenated as above) for maintenance and recording of composition: 124 mM NaCl, 3 mM KCl, 24 mM NaHCO3, 1.25 mM NaH2PO4, 1 mM MgSO4, 10 mM glucose, 2 mM CaCl2 (2 mM) and supplemented with 0.0001% gentamicin (Sigma-Aldrich, UK). Slice were rested at room temperature for ~ 20 min prior to transfer to recording chamber where they were further equilibrated for 1–2 h prior to the start of electrophysiological data collection. Where relevant (Fig. 6), ventral portions of the slice containing the SCN were removed by a scalpel cut (placed laterally ~ 400 μm dorsal to the optic chiasm and performed with the aid of a dissecting scope).

#### Acute electrophysiological recordings

Brain slices (prepared as above) were transferred to a submerged slice chamber (BSC-1; Digitimer Ltd., Welwyn Garden City, UK) and held in position by a weighted harp (ALA Scientific Instruments, NY, USA). The slice chamber was continuously perfused with oxygenated aCSF at a rate of ~ 1.5 ml/min via a peristaltic pump (120S Watson-Marlow; Falmouth, UK), with bath temperature maintained at ~ 33 °C via a PTC03 temperature controller (Digitimer Ltd.). A concentric bipolar stimulating electrode (FHC, ME, USA) was placed just above the optic chiasm between the paired SCN or, in a subset of experiments (*n* = 9), on one of the optic nerves. Two Silicon substrate 32-channel electrodes (NeuroNexus, MI, USA) were then positioned within the slice as appropriate with the aid of a dissecting microscope and M330 micromanipulators (World Precision Instruments, Hitchin, UK). For most experiments (*n* = 50/82), we positioned two Buszaki32L electrodes (4 shanks spaced 200 μm each with 8 closely-space recording sites) symmetrically close to the midline to target the SPZ, PVN and ventral thalamus (Fig. 1a). In some cases (*n* = 16), we instead used 8 × 1 tetrodes (8 shanks spaced 200 μm each with 4 closely-space recording sites) positioned in a similar manner to extend coverage of the SPZ and ventral thalamus. Further, in another subset of recordings (*n* = 16), we used a single Buszaki32L electrode to target the SPZ, PVN and ventral thalamus of one hemisphere and placed a single shank polytrode into the SCN (32sites spaced 25 μm in Poly3 configuration with a 105 μM core fibre terminating above the dorsal-most recording site; Fig. S3a). In all cases, following probe positioning and slice equilibration, neural signals were acquired by a SmartBox system (NeuroNexus) at 20KHz. Multiunit spiking detected at each electrode was monitored online to ensure effective stimulus delivery and slice responsiveness. Spike extraction and single unit isolation was then performed offline on virtual tetrode waveforms (regardless of multiunit responses apparent at that group of channels) as described previously [56] using custom Matlab (Mathworks, MA, USA) scripts and Offline Sorter V3 (Plexon Inc., TX, USA). Across all experiments, this resulted in 9.8 ± 0.7 (mean ± SEM) well-isolated units per 64 channel recording.

#### Long-term electrophysiological recordings

Brain slices were aligned on perforated multielectrode arrays (Multichannel Systems, MCS, Germany) arranged in a 6 × 10, 100 μm, grid comprising 59 active recording sites that spanned the SPZ, PVN and ventral thalamus (Fig. 5a). Slice position and placement of a concentric bipolar stimulating electrode was visualised under white light trans-illumination using a GXCAM-1.3 camera attached to a dissecting microscope (GX optical, UK). Within the recording chamber both slice surfaces were constantly perfused (flow rate ~ 3 ml/min) with oxygenated aCSF (33 °C ± 1 °C) via a combination of a constant vacuum pump (MCS gmbH, Germany) and a peristaltic pump (120S Watson-Marlow). Slice position was maintained using a weighted harp (ALA Scientific Instruments), alongside the suction caused by the flow of aCSF through the perforated MEA array. Following slice equilibration, neural activity was acquired continuously over > 26 h using MC_Rack software via a USB-ME64 system and MEA1060UP-BC amplifier (MCS GmbH, Germany). Signals were sampled at 50 kHz and high pass filtered at 200 Hz (Second order Butterworth). Spikes crossing a threshold (normally set at − 16.5 μV) were then extracted as timestamped waveforms (1.5 ms duration) and single unit activity was isolated offline by principle components based spike sorting using Offline Sorter V3 (Plexon Inc.), resulting in 20.9 ± 1.5 (mean ± SEM) well-isolated cells per recording.

#### Electrical and optogenetic stimulation

Electrical stimulation of the SCN region (or where relevant optic nerve) was delivered via concentric stimulating electrodes (FHC SKU30202; 25 μm/125 μm inner/outer pole diameter) coupled to a DS3 Isolated Constant Current Stimulator (Digitimer) or STG4004 stimulus generator (MCS) for acute and long-term recordings respectively. In both cases, stimuli were 300 μA, 200μS dipolar pulses and were delivered with an interstimulus interval of 2 s (acute recording) or 60 s (long-term recordings). For experiments using optogenetic stimulation in most cases, stimuli were delivered via a 200 μm core, 0.66NA, fibre positioned ~ 200 μm above the slice surface. The fibre was coupled to a 465 nm PlexBright table top module (Plexon Inc.) and 10 ms light pulses (~ 800 mW/mm^2^ at fibre tip) were interleaved with electrical pulses as above (i.e. optogenetic stimuli occurred 1 s or 30s after electrical stimulation for acute and long-term recordings). In a subset of recordings (Fig. S3), optogenetic stimuli were delivered locally to one SCN hemisphere via a 105 μm core 0.66NA fibre resting on the slice surface (delivering ~ 600 mW/mm^2^ at fibre tip).

#### Pharmacological manipulation

Pharmacological separation of the synaptic components of evoked responses was achieved by bath application of ionotropic glutamate receptor blockers (iGlu-X): NMDA (D-(-)-2-Amino-5-phosphonopentanoic acid; D-AP5, 50 μM) and AMPA/kainate (6-Cyano-7-nitroquinoxaline-2,3-dione disodium; CNQX, 20 μM). Contributions of GABAergic transmission were evaluated by bath perfusion with (+)-bicuculline (BIC, 20 μM), an antagonist at ionotropic GABA_A_ receptors which exhibits negligible activity at SK channels at the working concentration [57]. In most cases, after 1 h baseline recording slices were perfused with BIC for 30 min followed by BIC+iGlu-X for a further 30 min. In a subset of experiments (*n* = 13), slices received 30 min iGlu-X alone followed by 45 min washout before the drug application protocol outlined above. At the end of all experiments, slices were treated with bath application of *N*-Methyl-D-aspartic acid (NMDA, 20 μM) to confirm maintained cell responsiveness, followed by tetrodotoxin citrate (TTX, 1 μM), to confirm that acquired signals exclusively reflected Na^+^-dependent action potentials. All drugs were purchased from Sigma-Aldrich (UK) or Tocris (UK), dissolved in ddH_2_O and kept as stock solutions at − 20 °C (with the exception of BIC which was dissolved in DMSO) and were diluted to their respective final concentrations in pre-warmed, oxygenated aCSF.

### Data analysis

Acute changes in neural activity evoked by electrical or optogenetic stimuli were classified as excitatory or inhibitory when the average spike counts across multiple stimulus repeats (≥ 750) respectively exceeded the upper or lower bounds of the 99% confidence limits for prestimulus spike counts (NeuroExplorer v4; Nex technologies, CO, USA). To assess synaptic contributions to synaptically driven responses, within each relevant experimental block (25 min epochs prior to and during drug application), we calculated the maximum absolute stimulus-driven changes in spike firing occurring within 100 ms post-stimulus (25 ms moving window), relative to the mean baseline firing rate. Responses were subsequently compared by mixed-effects linear model with the slice in which the cell was isolated as a random factor and treatment (and, where relevant, time of day) as fixed factors with repeated measures as appropriate, followed by Sidak’s post-tests (SPSS v. 25, IBM, NY, USA). For analysis of latency, we calculated peri-stimulus histograms of spike count (15 ms moving window) and determined the first post-stimulus bin where spike counts significantly differed from the 5% confidence limits of the baseline spike count distribution. Subsequent statistical analysis was performed mixed-effects linear model including slice preparation as a random factor, as above. Comparisons of proportions of responding cells were performed using *χ*^2^ test or Fisher’s exact test as appropriate (GraphPad Prism 7, GraphPad Software Inc., CA, USA).

Analysis of long-term pMEA recordings was performed using Matlab routines, as described previously [18, 19]. Recorded neurons were considered to exhibit circadian variation when firing rate profiles over ≥ 26 h were better fit by a sinusoidal function (constrained to a periodicity of 20–28 h) than by a first order polynomial. For rhythmic channels, the projected ZT of peak firing was then determined from a 60s binned time-series (smoothed with a 2 h boxcar filter). Peak width and peak-trough amplitude were determined from this smoothed time-series, with the former representing the duration over which cell firing was > 50% of the peak within a single 24 h cycle. Comparisons between groups of neurons were performed by mixed-effects linear model with the slice in which each cell was detected as a random factor and cell type (and where relevant location) as fixed factors (SPSS v. 25). For analysis of cell phasing, we binned the time of peak firing for each cell in 6 h windows as a function of projected ZT or time since start of recording and tested for departure from a uniform distribution by *χ*^2^ test (GraphPad Prism). To account for variability in the number of identified cells of each type between different slices, we additional calculated, for each slice, the proportion of identified cells within each class with peak firing occurring in 6 h epochs as above. The latter data (shown in Figs. S6d,g and S7c) were analysed via mixed-effects linear models, with post hoc one-sample *t* tests for departure from a uniform distribution across bins as appropriate.

For analyses relating to anatomical distributions, the anatomical locations of neurons were estimated based on the distance between the recording site at which that cell was detected and a reference point located at the intersection of lines passing through the ventral boundary of the SCN and the midline. For acute recordings (where there was minimal variation in recording site position on the medial-lateral axis), cells were binned based on their projected dorsal-ventral location in the slice. For long-term pMEA recordings, cells we categorised as located within the SPZ, PVN or ventral thalamus based on projected locations determined as above and anatomical locations of the respective regions from [58].

## Supplementary information

**Additional file 1: Fig. S1.** Stimulating electrode positions for SCN and optic nerve activation.

**Additional file 2: Fig. S2.** Local electrical stimulation directly activates many SCN neurons.

**Additional file 3: Fig. S3.** Target cell responses to SCN stimulation do not exhibit substantial time of day variation.

**Additional file 4: Fig. S4.** Effect of ionotropic glutamate receptor antagonists on inhibitory responses to SCN and optic nerve stimulation.

**Additional file 5: Fig. S5.** Optogenetic activation of SCN GABA neurons drives inhibitory responses in downstream neurons.

**Additional file 6: Fig. S6.** Evoked and spontaneous activity of SCN target cells is unrelated to time of slice preparation or anatomical location.

**Additional file 7: Fig. S7.** Timing and amplitude of SCN-independent neuronal activity rhythms in hypothalamic and thalamic target structures ex vivo.

**Additional file 8.** Raw data used for statistical analysis in all main figures.

**Additional file 9.** Raw data used for statistical analysis in all supplementary figures.

## Data Availability

All data generated or analysed during this study are included in this published article and its Additional files. Raw data used for statistical analysis throughout the manuscript is available in Additional files 8 and 9.
