# Supplementary material for: Suprachiasmatic nucleus-dependent and independent outputs driving rhythmic activity in hypothalamic and thalamic neurons
Source: BMC Biol. 2020 Sep 30;18:134. doi: 10.1186/s12915-020-00871-8 (PMC7528611; doi:10.1186/s12915-020-00871-8)
Supplement: Supplementary file 1 — Additional file 1: Fig. S1. Stimulating electrode positions for SCN and optic nerve activation. [file 12915_2020_871_MOESM1_ESM.pdf]

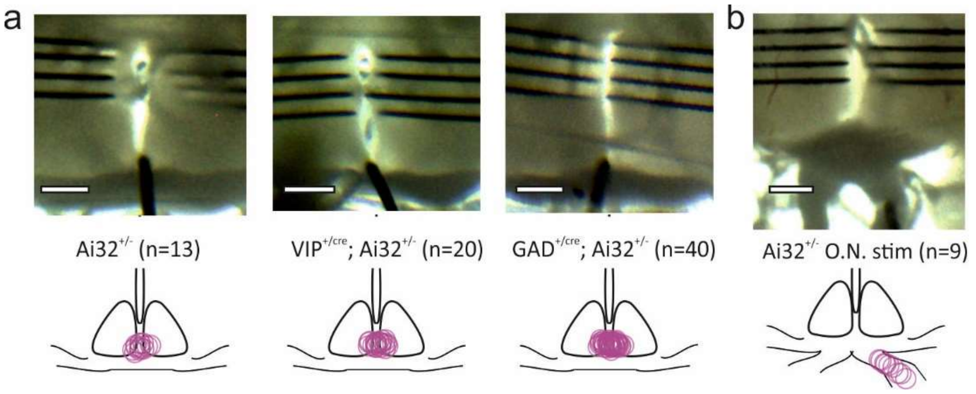

Figure S1. Stimulating electrode positions for SCN and optic nerve activation. (**a**) Top panels show in situ slice images of electrode placements from representative experiments from where the stimulating probe was placed in the SCN region (left top right: $Ai32^{+/-}$, $VIP^{+/cre}$;$Ai32^{+/-}$, $GAD^{+/cre}$; $Ai32^{+/-}$ slice). Bottom panels show overlay of SCN stimulating probe recording placements from all experiments in each genotype. (**b**) As above for experiments involving optic nerve stimulation. Scale bars throughout represent 500μm.
