# Supplementary material for: Suprachiasmatic nucleus-dependent and independent outputs driving rhythmic activity in hypothalamic and thalamic neurons
Source: BMC Biol. 2020 Sep 30;18:134. doi: 10.1186/s12915-020-00871-8 (PMC7528611; doi:10.1186/s12915-020-00871-8)
Supplement: Supplementary file 2 — Additional file 2: Fig. S2. Local electrical stimulation directly activates many SCN neurons. [file 12915_2020_871_MOESM2_ESM.pdf]

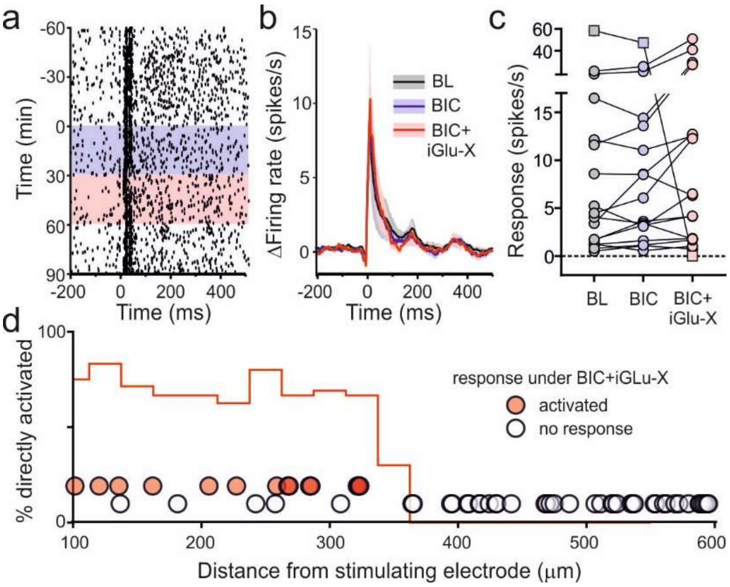

Figure S2. Local electrical stimulation directly activates many SCN neurons. (**a**) Peri-stimulus raster plot for a representative SCN neuron in response to local electrical stimulation (as in Fig 1) before and after treatment with ionotropic GABA (BIC; 20µm (+)-bicuculline) and Glutamate antagonists and (iGlu-X; 50µm D-AP5 and 20µm CNQX). (**b**) Mean±SEM change in firing for SCN neurons responding to local electrical stimulation before and after treatment with ionotropic GABA and Glutamate antagonists (n=17/23 neurons recorded from SCN region). (**c**) Mean response (peak change in firing occurring within 100ms following stimulation) across 17 SCN neurons with excitatory responses to SCN stimulation before and after treatment with ionotropic GABA and Glutamate antagonists. Note that all but one cell maintains responses under BIC+iGlu-X. (**d**) Distribution of cells showing direct (BIC+iGlu-X-insensitive) activation following local SCN stimulation as a function of linear distance from the stimulating electrode. Circles show individual cells, histogram shows percentage of identified cells within a 100µm moving window.
