# Supplementary material for: Suprachiasmatic nucleus-dependent and independent outputs driving rhythmic activity in hypothalamic and thalamic neurons
Source: BMC Biol. 2020 Sep 30;18:134. doi: 10.1186/s12915-020-00871-8 (PMC7528611; doi:10.1186/s12915-020-00871-8)
Supplement: Supplementary file 3 — Additional file 3: Fig. S3. Target cell responses to SCN stimulation do not exhibit substantial time of day variation. [file 12915_2020_871_MOESM3_ESM.pdf]

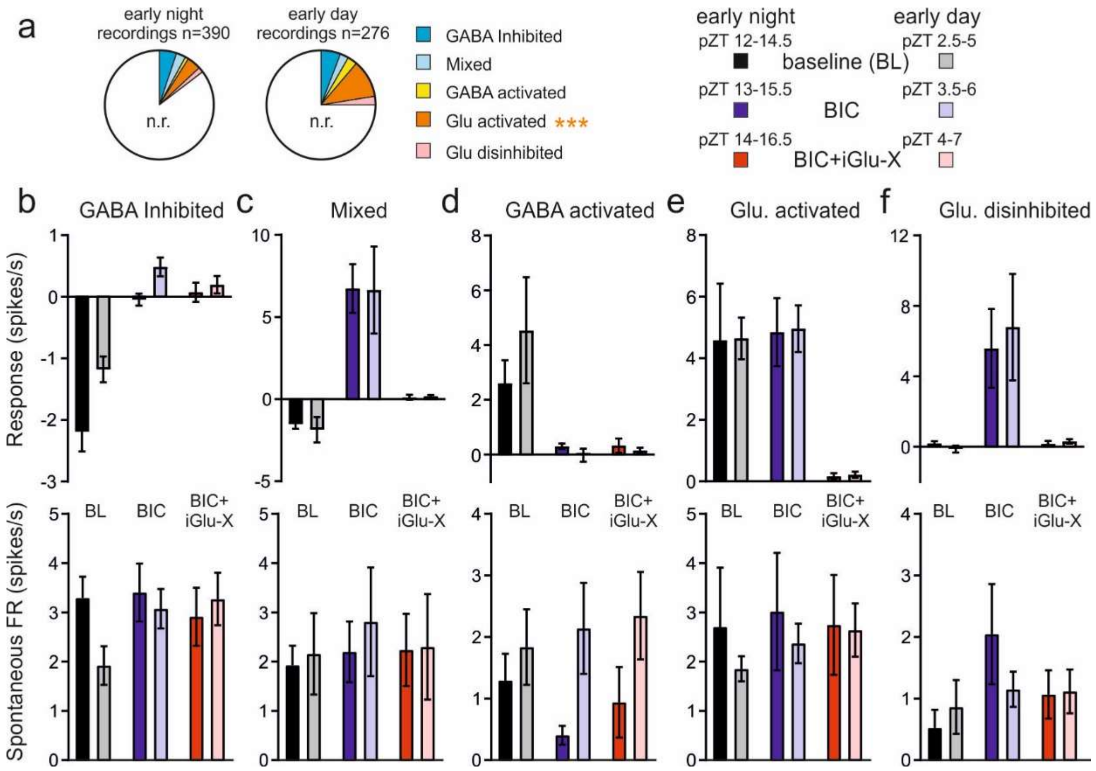


**Figure S3. Target cell responses to SCN stimulation do not exhibit substantial time of day variation.** (**a**) Proportions neurons exhibiting the various identified classes of response to SCN stimulation in slices recorded during early portions of the projected night and day (projected Zeitgeber times; pZT indicated to right). Data analysed by $\chi^2$-test (P=0.003) followed by Fisher's exact tests for proportions of each individual cell class (all P>0.05 except Glu. activated, P=0.0006). (**b-f**) Upper panels show mean±SEM response of cells from each class in the presence and absence of ionotropic GABA and glutamate antagonists from early night and early day recordings. Lower panels show corresponding spontaneous firing rates of the cells under each condition. Data analysed by mixed effects linear model. The effects of drug treatment (upper panels) did not vary significantly as a function of time of day for any cell type (**b**: Treatment- $F_{2,49.5}$=38.9, P<0.001; pZT- $F_{1,13.9}$=9.2, P=0.01, pZT X Treatment- $F_{2,49.5}$=2.3, P=0.11; **c**: Treatment- $F_{2,17.3}$=23.8, P<0.001; pZT- $F_{1,18.2}$=0.2, P=0.90, pZT X Treatment- $F_{2,17.3}$=0.1, P=0.87; **d**: Treatment- $F_{2,10.6}$=2.9, P=0.1; pZT- $F_{1,10.7}$=0.3, P=0.63, pZT X Treatment- $F_{2,10.6}$=0.4, P=0.71; **e**: Treatment- $F_{2,45.7}$=39.9, P<0.001; pZT- $F_{1,90.2}$=0.0, P=0.93, pZT X Treatment- $F_{2,45.7}$=0.0, P=0.99; **f**: Treatment- $F_{2,18.1}$=6.0, P=0.01; pZT- $F_{1,11.6}$=0.1, P=0.76, pZT X Treatment- $F_{2,18.1}$=1.4, P=0.28). Baseline firing rates (lower panels) were not significantly influenced by treatment or time of day for any cell type (**b**: Treatment- $F_{2,53.5}$=1.7, P=0.19; pZT- $F_{1,20.6}$=0.1, P=0.78, pZT X Treatment- $F_{2,53.5}$=2.8, P=0.07; **c**: Treatment- $F_{2,22}$=0.5, P=0.64; pZT- $F_{1,12.7}$=0.0, P=0.92, pZT X Treatment- $F_{2,22}$=0.4, P=0.69; **d**: Treatment- $F_{2,17}$=0.1, P=0.88; pZT- $F_{1,8.5}$=4.2, P=0.07, pZT X Treatment- $F_{2,17}$=0.4, P=0.68; **e**: Treatment- $F_{2,67.7}$=1.0, P=0.39; pZT- $F_{1,20.6}$=0.6, P=0.45, pZT X Treatment- $F_{2,67.7}$=0.4, P=0.67; **f**: Treatment- $F_{2,19.6}$=2.1, P=0.16; pZT- $F_{1,7.4}$=0.0, P=0.87, pZT X Treatment- $F_{2,19.7}$=0.9, P=0.42).
