# Supplementary material for: Suprachiasmatic nucleus-dependent and independent outputs driving rhythmic activity in hypothalamic and thalamic neurons
Source: BMC Biol. 2020 Sep 30;18:134. doi: 10.1186/s12915-020-00871-8 (PMC7528611; doi:10.1186/s12915-020-00871-8)
Supplement: Supplementary file 4 — Additional file 4: Fig. S4. Effect of ionotropic glutamate receptor antagonists on inhibitory responses to SCN and optic nerve stimulation. [file 12915_2020_871_MOESM4_ESM.pdf]

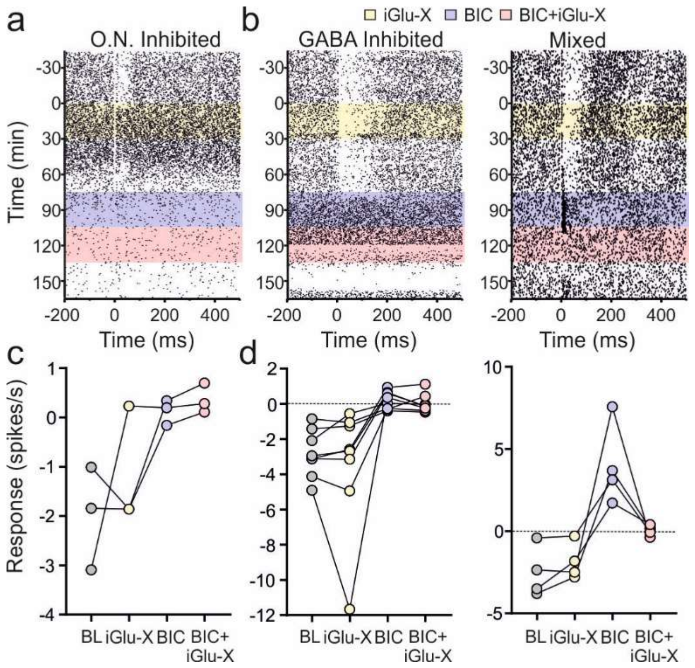

Figure S4. Effect of ionotropic glutamate receptor antagonists on inhibitory responses to SCN and optic nerve stimulation. (**a**, **b**) Representative peri-stimulus spike rasters for cells exhibiting inhibitory responses to optic nerve stimulation (**a;** O.N. inhibited) or SCN stimulation (**b**), treated with inotropic glutamate (iGlu-X; 50µm D-AP5 and 20µm CNQX) and/or GABA receptor antagonists (BIC; 20µm (+)-bicuculline). (**c**,**d**) Responses of all identified neurons showing inhibitory responses to optic nerve stimulation (**c**) and 'GABA inhibited' and 'Mixed' responses to SCN stimulation (**d**). Note that for one of three O.N. inhibited cells (cell shown in panel **a**), inhibitory responses were blocked by iGlu-X alone whereas as this did not occur for any cells exhibiting inhibitory responses to SCN stimulation.
