# Supplementary material for: Suprachiasmatic nucleus-dependent and independent outputs driving rhythmic activity in hypothalamic and thalamic neurons
Source: BMC Biol. 2020 Sep 30;18:134. doi: 10.1186/s12915-020-00871-8 (PMC7528611; doi:10.1186/s12915-020-00871-8)
Supplement: Supplementary file 5 — Additional file 5: Fig. S5. Optogenetic activation of SCN GABA neurons drives inhibitory responses in downstream neurons. [file 12915_2020_871_MOESM5_ESM.pdf]

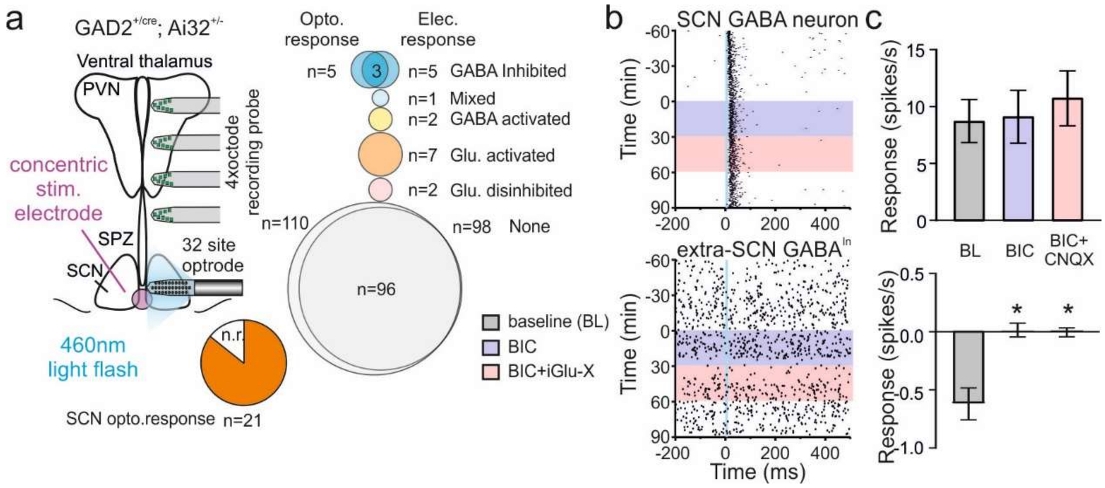

Figure S5. Optogenetic activation of SCN GABA neurons drives inhibitory responses in downstream neurons. (**a**) Left shows a schematic of the recording configuration for experiments using SCN electrical and optogenetic stimulation in $GAD2^{+/cre}$; $Ai32^{+/-}$ slices, with local recording and optogenetic stimulation of one SCN hemisphere via a 32-site optrode. Right plot illustrates proportions of responding cells and their overlap following optical and electrical stimulation (bubble size proportional to population sizes). Lower pie chart shows proportion of isolated SC neurons with excitatory response to optogenetic stimulation (n.r. indicates no response). (**b**) peri-stimulus spike rasters for representative SCN neuron showing direct excitatory response to optogenetic stimulation (upper) and extra-SCN neuron with synaptically mediated inhibitory responses ($GABA^{In}$; lower panel), under baseline conditions and following $GABA_A$ receptor blockade (BIC; 20µm (+)-bicuculline) in the absence and presence of ionotropic glutamate receptor antagonists (iGlu-X; 50µm D-AP5 and 20µm CNQX). (**c**) Mean±SEM responses of SCN GABA neurons (upper; n=18) and extra-SCN $GABA^{In}$ cells (lower; n=5) before and after antagonist treatment. Data analysed by mixed-effects linear model (upper: $F_{2, 31.3}$=0.3, P=0.76; lower: $F_{2, 5.5}$=9.9, P=0.02) with Sidak's post-tests. *=P<0.05.
