# Supplementary material for: Suprachiasmatic nucleus-dependent and independent outputs driving rhythmic activity in hypothalamic and thalamic neurons
Source: BMC Biol. 2020 Sep 30;18:134. doi: 10.1186/s12915-020-00871-8 (PMC7528611; doi:10.1186/s12915-020-00871-8)
Supplement: Supplementary file 6 — Additional file 6: Fig. S6. Evoked and spontaneous activity of SCN target cells is unrelated to time of slice preparation or anatomical location. [file 12915_2020_871_MOESM6_ESM.pdf]

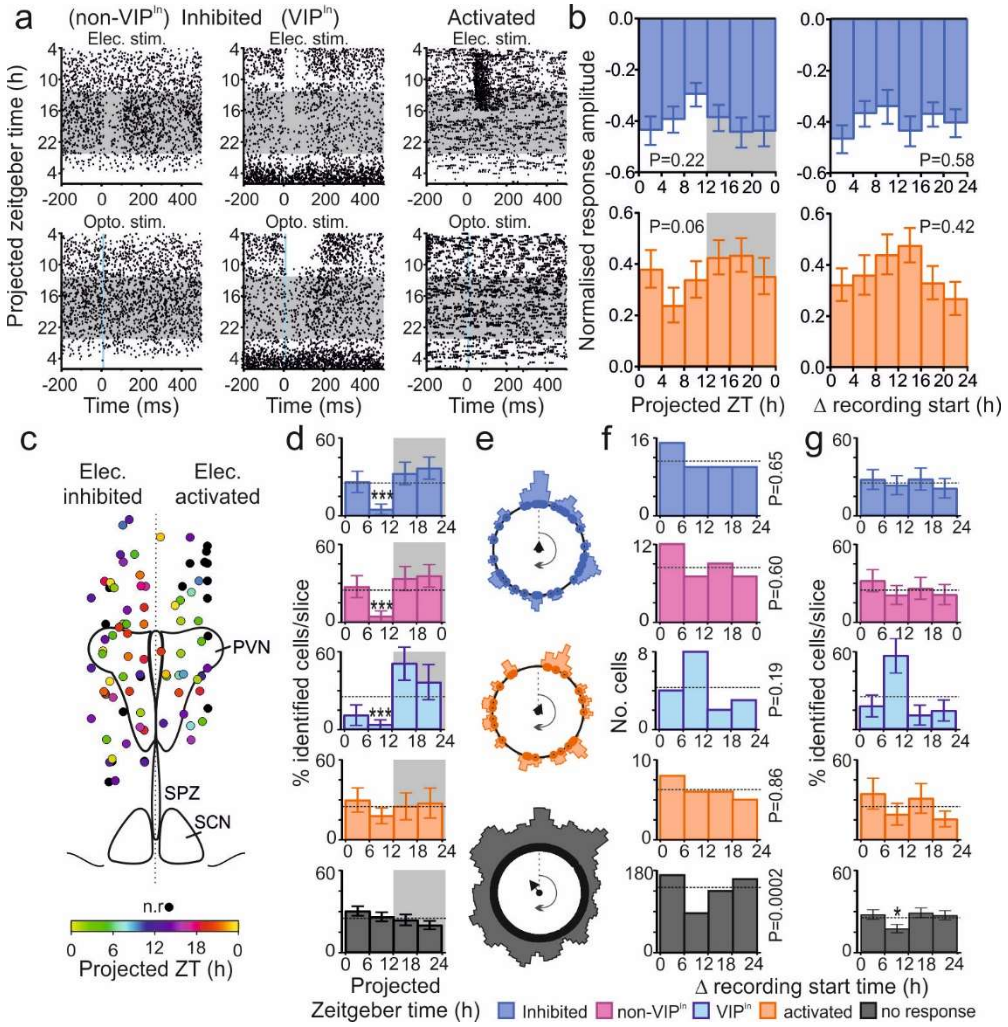

Figure S6. Evoked and spontaneous activity of SCN target cells is unrelated to time of slice preparation or anatomical location. (**a**) Peri-stimulus rasters for electrical (upper) and optogenetic SCN stimulation over 26h, illustrating cells with inhibitory responses to electrical stimulation that did or did not receive inhibitory input from SCN VIP cells (left and mid respectively) and a neuron with excitatory response to electrical stimulation (right). (**b**) Mean±SEM responses of cells that were inhibited (upper; n=54) or activated (lower; n=35) by electrical stimulation of the SCN region, expressed as a function of projected Zeitgeber time (left) or time since start of recording (right). Data analysed by mixed effects linear model (top left: $F_{5,200}$=1.43, P=0.22; top right: $F_{5,105}$=0.76, P=0.58; bottom left: $F_{5,56.9}$=2.25, P=0.06; bottom right: $F_{5,59.1}$=1.00, P=0.42). (**c**) Estimated anatomical locations of neurons showing inhibition or activation following electrical stimulation of the SCN, colour coded according to phase of peak firing (cells classed as non-rhythmic shown in black; n.r.).(**d**) Mean±SEM percentage of identified cell types in each slice with peak firing during specific epochs relative to animal's prior LD cycle. Data analysed by mixed-effects linear model (Inhibited: $F_{3,49.9}$=6.17, P=0.001; Non-VIP^{In}: $F_{3,48.2}$=6.01, P=0.001; VIP^{In}: $F_{3,21.6}$=5.53, P=0.006; Activated: $F_{3,24.6}$=0.50, P=0.69; No response: $F_{3,63.7}$=1.70, P=0.18) with post-hoc one-sample t-tests vs. proportion expected for a uniform distribution. (**e**) Rayleigh vector plots for identified cell populations (as in Fig. 5g) showing distributions of peak times relative to start of recording. (**f**) Histograms of cell counts for identified cell populations as a function of time since start of recording Data compared vs. a uniform distribution by χ^2-test. (**g**) Analysis as in d, binned relative to start of recording. Data analysed by mixed-effects linear model (Inhibited: $F_{3,46.1}$=0.20, P=0.90; Non-VIP^{In}: $F_{3,45}$=0.41, P=0.75; VIP^{In}: $F_{3,30.1}$=3.07, P=0.04; Activated: $F_{3,28.7}$=1.02, P=0.40; No response: $F_{3,61.6}$=2.84, P=0.045). *, *** = p<0.05 and P<0.001 respectively.
