# Supplementary material for: Suprachiasmatic nucleus-dependent and independent outputs driving rhythmic activity in hypothalamic and thalamic neurons
Source: BMC Biol. 2020 Sep 30;18:134. doi: 10.1186/s12915-020-00871-8 (PMC7528611; doi:10.1186/s12915-020-00871-8)
Supplement: Supplementary file 7 — Additional file 7: Fig. S7. Timing and amplitude of SCN-independent neuronal activity rhythms in hypothalamic and thalamic target structures ex vivo. [file 12915_2020_871_MOESM7_ESM.pdf]

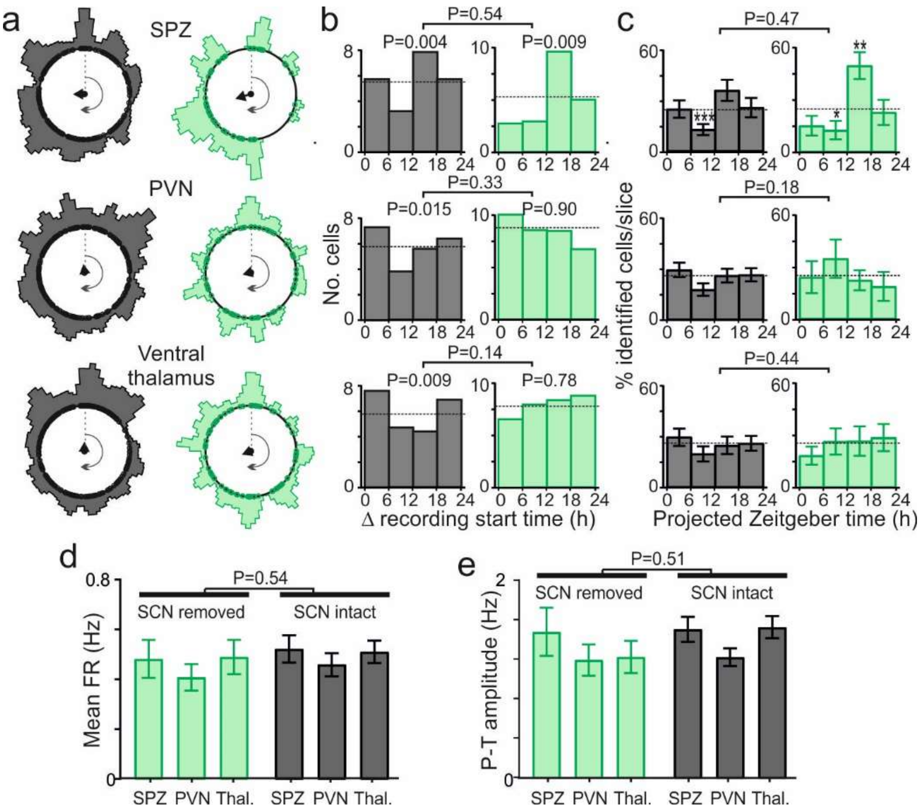

Figure S7. Timing and amplitude of SCN-independent neuronal activity rhythms in hypothalamic and thalamic target structures ex vivo. (**a**) Rayleigh vector plot of time of peak firing for rhythmic SPZ (top), PVN (mid) and ventral thalamic neurons (bottom) in slices containing (left) or lacking and SCN (right), plotted relative to time since start of recording. Outer histogram smoothed with Gaussian, SD=15min; individual neurons represented by inner dots. (**b**) Cell counts for populations in **a** across 6h bins as a function of time since start of recording as in. Data compared vs. a uniform distribution and between SCN containing and lacking slices by χ^2-tests. Equivalent analysis performed as a function of projected Zeitgeber time of peak firing did not reveal departure from uniformity in any group (χ^2-tests all P>0.05). (**c**) Data from **b**, expressed as mean±SEM percentage of identified populations in each slice with peak firing during specific epochs relative to recording start. Data analysed by mixed-effects linear model (SPZ: Epoch-$F_{3,75.8}$=7.30, P<0.001; SCN-$F_{1,137}$=0.0, P~1.0; Epoch X SCN- $F_{3,75.8}$=0.86, P=0.47; PVN: Epoch-$F_{3,79.1}$=0.02, P=0.89; SCN-$F_{1,156}$=0.0, P~1.0; Epoch X SCN- $F_{3,79.1}$=1.69, P=0.18; Thalamus: Epoch-$F_{3,82.6}$=0.21, P=0.89; SCN-$F_{1,169}$=0.0, P~1.0; Epoch X SCN- $F_{3,82.6}$=0.91, P=0.44), with post-hoc one-sample t-tests vs. proportions expected for a uniform distribution. (**d**) Mean±SEM 24h average firing rate for rhythmic neurons isolated from SCN target regions in slices lacking or containing the SCN. Data analysed mixed-effects linear model (SCN: $F_{1,47.1}$=0.38, P=0.54; Region: $F_{2,680}$=0.81, P=0.45; Interaction: $F_{2,680}$=0.01, P=0.99). (**e**) Mean±SEM peak-trough amplitude of firing rate rhythms for neurons isolated from SCN target regions in slices lacking or containing the SCN. Data analysed by two-way ANOVA (SCN: $F_{1,49.6}$=0.45, P=0.51; Region: $F_{2,713}$=1.50, P=0.22; Interaction: $F_{2,713}$=0.34, P=0.71). *,**,***=P<0.05, P<0.01 and P<0.001 respectively.
